# Cross-continental comparison of the association between the physical environment and active transportation in children: a systematic review

**DOI:** 10.1186/s12966-015-0308-z

**Published:** 2015-11-26

**Authors:** Sara D’Haese, Griet Vanwolleghem, Erica Hinckson, Ilse De Bourdeaudhuij, Benedicte Deforche, Delfien Van Dyck, Greet Cardon

**Affiliations:** Faculty of Medicine and Health Sciences, Department of Movement and Sports Sciences, Ghent University, Watersportlaan 2, 9000 Ghent, Belgium; Centre for Physical Activity and Nutrition Research, Auckland University of Technology, Auckland, New Zealand; Faculty of Medicine and Health Sciences, Department of Public Health, Ghent University, De Pintelaan 185, 9000 Ghent, Belgium; Faculty of Physical Education and Physiotherapy, Department of Human Biomechanics and Biometry, Vrije Unversiteit Brussel, Pleinlaan 2, 1050 Brussel, Belgium; Research Foundation Flanders (FWO), Egmontstraat 5, 1000 Brussels, Belgium

**Keywords:** Walkability, Walk/cycle facilities, Aesthetics, Safety, Recreation facilities, Active travel, Walking, Cycling, Children

## Abstract

**Background:**

The purpose of this systematic review was to determine the relationship between a wide range of physical environmental characteristics and different contexts of active transportation in 6- to 12-year-old children across different continents.

**Methods:**

A systematic search was conducted in six databases (Pubmed, Web of Science, Cinahl, SportDiscus, TRIS and Cochrane) resulting in 65 papers, eligible for inclusion. The investigated physical environmental variables were grouped into six categories: walkability, accessibility, walk/cycle facilities, aesthetics, safety, recreation facilities.

**Results:**

The majority of the studies were conducted in North America (*n* = 35), Europe (*n* = 17) and Australia (*n* = 11). Active transportation to school (walking or cycling) was positively associated with walkability. Walking to school was positively associated with walkability, density and accessibility. Evidence for a possible association was found for traffic safety and all forms of active transportation to school. No convincing evidence was found for associations between the physical environment and active transportation during leisure.

General safety and traffic safety were associated with active transportation to school in North America and Australia but not associated with active transportation to school in Europe.

**Conclusions:**

The physical environment was mainly associated with active transportation to school. Continent specific associations were found, indicating that safety measures were most important in relation to active commuting to school in North America and Australia. There is a need for longitudinal studies and studies conducted in Asia, Africa and South-America and studies focusing specifically on active transportation during leisure.

**Electronic supplementary material:**

The online version of this article (doi:10.1186/s12966-015-0308-z) contains supplementary material, which is available to authorized users.

## Background

Despite the numerous health benefits of daily physical activity (=PA), there is evidence of decreasing PA levels in children [1]. As a high level of PA in children predicts a high level of adult PA, it is important to promote PA during childhood [2]. Therefore, insight into determinants of children’s PA is necessary for developing effective interventions.

Although young people report a preference to be active, they are often limited by external factors like parental rules and physical environmental factors [1, 3]. According to ecological models, environmental factors (e.g. physical, political, economic or sociocultural environment) can influence behaviors directly as well as indirectly by influencing self-efficacy, attitude, subjective norm, perceived behavioral control or intention [4]. As PA is performed in physical settings (e.g. the physical environment), it is important to further explore environmental correlates.

Reviews concerning physical environmental determinants of active transportation in adults and adolescents found associations between several aspects of the physical environment and PA [5–8]. Compared to adults and adolescents whose independent mobility reaches beyond their own neighborhood, 6- to 12-year-old children’s independent mobility is usually restricted to their own neighborhood environment [9]. Children also have different behavioral patterns and are not permitted to drive motorized vehicles. Therefore, the association between the physical environment and active transportation should be studied in this specific age group.

Few reviews on environmental correlates of active transportation in children have been published. In most reviews concerning the association between the physical environment and PA, specific physical environmental topics (e.g. safety [10]) were studied in a broader age-range [11, 12] in relation to overall PA [13] or moderate- to vigorous-intensity physical activity [14]. However some researchers presume that the relationship between the physical environment and PA may vary according to the domain of the activity (e.g. active transport to school, active transportation during leisure, moderate- to vigorous-intensity PA,…) [13, 15]. As environmental correlates can have a dissimilar impact on different domains and contexts of PA [13, 16, 17], it is necessary to study the influence of the physical environment separately on specific domains of PA [13, 18]. As it is hypothesized that the influence of children’s physical neighborhood environment on PA at school or in the sports club may be limited, the predictive capacity of physical environmental correlates of PA may improve if PA that takes place in the neighborhood (e.g. active transportation) is studied in relation to the physical environment, rather than overall PA.

Moreover, in physical environment literature, it is often presumed that the relationship between the physical environment and PA differs across different continents and countries [8] as physical environmental attributes and PA behaviors are different across continents. For example, due to suburbanization and peripheral centers, most US cities are less dense compared to European cities [19]. Furthermore, many cities in Europe have grown by accretion rather than by urban planning, whereas in American cities, planned neighborhoods with city blocks and grid patterns are much more common, compared to Europe. Also transportation modes differ as, for example, average trip distances are usually shorter in Europe [20, 21] compared to North America and Australia. Despite differences across continents, no one has systematically investigated the relationship between the physical environment and children’s active transportation across different continents.

A first aim of this systematic review was to determine the association between a wide range of specific physical environmental characteristics (walkability, walk/cycle facilities, safety, aesthetics and recreation facilities) and different contexts of active transportation (active transportation to school and active transportation during leisure) in 6- to 12-year-old children. The second aim was to investigate this relation across different continents. This review is the first to provide a wide overview of physical environmental correlates of specific contexts of active transportation in children.

## Methods

### Search strategy

Articles were searched in the following six electronic databases: Pubmed, Web of Science, Cinahl, SportDiscus, TRIS and Cochrane and the following search terms were used: (determinant* OR correlate* OR influence* OR association* OR relat* OR associate*) AND (environment* OR physical OR built OR neighbor*hood OR facilit* OR walkab* OR playability OR urban design OR crime OR field* OR aesthetic* OR safe* OR equipment OR park OR playground OR recreation* OR land use mix OR residential OR connect*) AND (physical activity OR physically active lifestyle OR physically active OR leisure activities OR recreation OR walk* OR cycle* OR cycli* OR bik* OR transport* OR commut* OR active travel) AND (child* OR boy OR boys OR girl OR girls OR pupil* OR young* OR youth OR adolescent*) NOT (intervention OR comment OR disabled OR patients OR institutionalized).

**Fig. 1:**
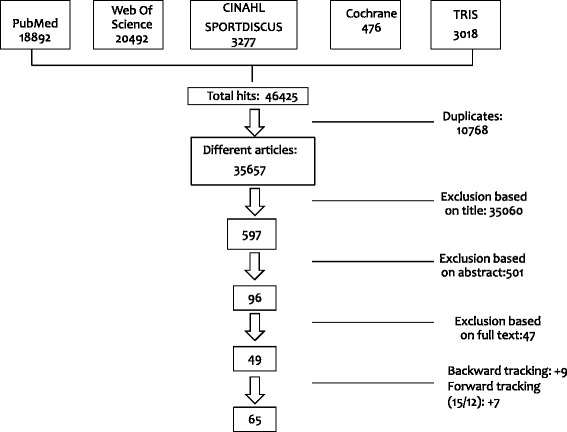
Flow chart of the systematic literature search

The search was limited to English articles published from January 2000 to August 2014. This time period was chosen, as most of the relevant literature was published during the last decade [13]. Moreover, environments are changing, therefore it is important to study only the most recent literature concerning the relation between environments and active transportation. The PRISMA guidelines [22] were followed to select the eligible articles. First, duplicates were excluded from the databases (*n* = 10768), afterwards; exclusions were first made on title (*n* = 35 060), than on abstract (*n* = 501) and finally on full text (*n* = 47). This resulted in 49 eligible articles. Backward (screening the reference lists of the included articles) and forward tracking (screening the citations of the included articles on Web of Science) resulted in 16 additional studies. A total of 65 studies were included in this review. A flowchart of the literature search is shown in Fig. 1. The literature search was conducted by the first author, but a second expert opinion was asked in case of doubt whether to include or exclude a study; the eligibility of doubtful studies was discussed until consensus was reached. The review is registered in PROSPERO, the International prospective register of systematic reviews (registration number: CRD42014013778).

### Variables

The physical environment was defined as objective and perceived characteristics of the physical context in which children spend their time (e.g. neighborhood, school, home) including aspects of urban design (e.g. presence and structure of sidewalks), traffic density and speed, distance to and design of venues for PA (e.g. playgrounds, parks and school yards), crime and safety [11].

Aspects of the physical environment were divided in the following categories [23]: 1) walkability, 2) accessibility, 3) walk/cycle facilities, 4) aesthetics, 5) safety and 6) recreation facilities. The physical environmental variables were categorized according to the categories represented in the Neighborhood Environment Walkability Scale (NEWS); the most frequently used environmental questionnaire [24].

Within these categories, different items were grouped together and subcategories were created as shown in the additional file (Additional file 1) to reduce the number of variables. Subcategories of walkability included residential density, street connectivity and land use mix diversity. When these categories were summed together to investigate the association with active transportation, they were categorized as ‘walkability’. Safety was subdivided into general safety, traffic safety and crime safety. Traffic safety consisted of items measuring safety aspects of the traffic situation such as the presence of traffic lights, speed bumps, traffic hazards and traffic volume. Crime safety consisted of the items measuring stranger danger, concerns of crime and violence. When safety was not further specified as traffic or crime safety (e.g. “it is safe to play outside”), items were classified under ‘general safety’.

As De Vries et al. showed that environmental correlates for active transportation differed according to purpose; active transportation was divided into two subcategories: active transportation to school and active transportation during leisure time [15]. Moreover, correlates for active transportation can differ according to commuting mode [15]. Therefore, active transportation in both categories was divided into walking and cycling when this was possible, resulting in six categories: walking to/from school (=WTS), cycling to/from school (=CTS), walking or cycling to/from school (=ATS), walking for transportation during leisure time, cycling for transportation during leisure time and walking or cycling for transportation during leisure.

### Selection criteria

Articles were included if they investigated the physical neighborhood environment in relation to any context of active transportation. The age of the children studied ranged from 6 to 12 year. When the mean age of the children was not mentioned in the study, the corresponding author of the study was contacted to ask for the mean age of the children. When the author did not reply (*n* = 5), studies were excluded. Intervention studies, systematic reviews, qualitative reports, expert opinions or conference proceedings were excluded. Studies investigating overweight, disabled or institutionalized children were also excluded.

### Data extraction

To review the characteristics of the included studies, detailed information on design, participants, methods and results were summarized into a supplementary table. This table is included in this review as Additional file 1. As it is possible that analyses in different articles were adjusted for different variables, unadjusted odds ratios were reported in order to avoid bias among different publications [25]. When unadjusted odds ratios were not available, results from the least adjusted analyses were reported. When analyses were conducted separately for boys and girls, “b” (boys) or “g” (girls) was indicated in superscript in Additional file 2. The percentage of associations between objective and perceived environmental characteristics and active transportation was calculated by dividing the number of positive relations between the physical environment and active transportation to school and during leisure in the expected direction, by the number of total investigated relationships for objective and subjective physical environmental characteristics.

### Coding associations

As it was expected that there would be large heterogeneity in the included studies concerning the methods of the physical environment measurement, type of physical activity measure that was used and the use of adjusting variables, an a priori decision was made, not to meta-analyze the data. Instead, a classification system similar to previous systematic reviews was used [26]. Each environmental characteristic received a summary code: + or -, indicating a positive/negative relationship, (+) or (−) indicating a relationship with evidence for a possible association and 0, indicating no relationship [26]. This summary code was based on the number of investigated associations. For example, if a study did not find a relationship between the presence of roundabouts and cycling to school and the same study did not find a relationship between presence of intersections and cycling to school; street connectivity was coded two times as ‘no relationship’; as roundabouts and intersections are both measures of street connectivity [15]. When less than 34 % of the investigated associations supported the association, it was deemed that there was no evidence to support the association. Variables showed evidence for a possible association if 34–59 % of the investigated associations supported the association. Convincing evidence was attributed to variables, when 60 % or more of the investigated associations supported the association [26]. Only variables that were studied in at least three different studies received a summary code.

### Quality assessment

To assess the quality of the included articles, the quality assessment tool ‘QUALSYST’ from the “Standard Quality Assessment Criteria for Evaluating Primary Research Papers from a Variety of Fields” (Alberta Heritage Foundation for Medical Research) was applied [27]. QUALSYST consists of 14 criteria that were scored on a 3-point scale (2 = yes, 1 = partially, 0 = no) depending on the degree to which the specific criteria were met [27]. When criteria were not applicable to a study design, these criteria were marked “non-applicable” and were excluded from the calculation of the summary score. The scores were summed and divided by the total number of items (excluding those non-applicable), to obtain a summary score for each paper. The score was then converted into a percentage of the maximum possible score. Two authors (SDH, GV) have reviewed the included papers for quality, and any discrepancies were further discussed to come to an agreement. Only studies with a quality score over 75 % were included in the tables to draw results concerning the association between the physical environment and physical activity [27].

## Results

### Study characteristics

A total of 65 studies were identified for this review. Study characteristics are presented in Table 1. Only four studies of the 65 used a longitudinal design [28–31]. More than half of the studies (*n* = 35) were conducted in North America [16, 23, 28, 32–63]; 17 studies were conducted in Europe [15, 29, 64–78]; 11 in Australia [30, 31, 79–87]; and two studies were conducted in Asia [88, 89]. In 32 studies, geographic information systems (GIS) were used to determine the environmental characteristics objectively [29, 31, 32, 34, 38, 40, 43, 45, 48–50, 52–55, 60, 61, 66, 67, 69, 72, 73, 76–80, 85–89]. All studies were published after 2003. The quality of the included studies ranged from 68.2 to 95.5 %. The quality of the studies was relatively high as only four studies were excluded from Tables 2 and 3 because they had a quality score under 75 %. The remaining studies had a quality score between 77.3 and 95.5 %. Within the included studies, lower scores were mainly obtained for the appropriateness of the study design, as most studies used a cross-sectional design.

**Table 1:** Methodological characteristics of the included studies

	Quality score	Study design		Sample size				Geographic area				PA measurement			Environment				
		Cross-sectional	Longitudinal	n ≤ 150	150 < n ≤ 500	500 < n ≤ 1000	n > 1000	North America	Europe	Australia	Asia	Parental report	Children’s report	Observation	Parental report	Children’s report	GIS/census	Audit data	School principal’s report
Aarts et al. [64]	86.4	x					x		x			x			x				
Alton et al. [65]	90.9	x			x				x				x		x	x			
Braza et al. [32]	72.7	x					x	x					x				x		
Bringolf-Isler et al. [66]	90.9	x					x		x			x			x		x		
Carson et al. [16]	86.4	x					x	x				x			x				
Carver et al. [79]	86.4	x			x					x		x					x		
Carver et al. [31]	95.5		x		x					x		x					x		
Chillon et al. [33]	86.4	x					x	x					x		x			x	
Christiansen et al. [67]	90.9	x					x		x				x			x	x		
Curreiro et al. [34]	81.8	x			x			x					x		x	x	x	x	
Cutumisu et al. [35]	86.4	x				x		x				x			x	x			
de Vries et al. [15]	90.9	x			x				x			x	x					x	
DeWeese et al. [36]	90.9	x				x		x				x			x				
D’Haese et al. [68]	95.5	x				x			x			x			x				
D'Haese et al. [69]	95.5	x			x				x			x					x		
Ducheyne et al. [70]	95.5	x				x			x			x			x				
Durand et al. [37]	95.5	x			x			x					x		x				
Frank et al. [38]	90.9	x				x		x				x					x		
Gallimore et al. [39]	68.2	x		?	?	?	?	x				?	?					x	
Giles-Corti et al. [80]	90.9	x					x			x		x					x		
He [40]	81.8	x					x	x					x				x		
Hsu and Saphores, [41]	81.8	x				x		x				?	?		x				
Hume et al. [30]	95.5		x	x						x		x			x				
Johansson [71]	77.3	x			x				x			x			x			x	
Kemperman and Timmermans [72]	81.8	x					x		x			x	x				x		
Kerr et al. [42]	95.5	x			x			x				x			x				
Kytta et al. [73]	90.9	x				x			x				x				x		
Larouche et al. [44]	68.2	x		x				x					x		x	x			
Larouche et al. [43]	95.5	x				x		x					x				x		x
Larsen et al. [45]	77.3	x				x		x				x			x	x	x	x	
Lee et al. [46]	90.9	x					x	x				x			x				
Leslie et al. [81]	86.4	x					x			x			x			x			
Lin & Chang [89]	72.7	x			x						x		x				x	x	
Lin & Yu [88]	77.3	x			x						x		x				x		
Loucaides et al. [74]	81.8	x			x				x				x			x			
Martin et al. [47]	95.5	x					x	x				x				x			
McDonald [48]	77.3	x				x		x				x					x		
McDonald [49]	86.4	x					x	x				x					x		
McDonald [50]	86.4	x					x	x				x					x		
McMillan [51]	81.8	x		?	?	?		x				x			x			x	
Merom et al. [82]	86.4	x				x				x		x			x				
Mitra et al. [54]	81.8	x					x	x				x					x		
Mitra and Buliung [53]	86.4	x					x	x				x					x		
Mitra and Buliung [52]	95.5	x				x		x				x					x		
Noland et al. [55]	86.4	x					x	x				x					x		
Napier et al. [56]	90.9	x			x			x					x						
Oluyomi et al. [57]	90.9	x				x		x				x			x	x			
Pabayo et al. [28]	95.5		x			x		x				x			x				
Page et al. [75]	86.4	x					x		x				x			x			
Panter et al. [77]	95.5	x					x		x			x	x				x	x	
Panter et al. [76]	95.5	x					x		x			x	x		x	x	x		
Panter et al. [29]	95.5		x			x			x				x		x	x	x		
Rodriguez and Vogt [58]	86.4	x					x	x					x			x			
Rosenberg et al. [23]	90.9	x		x				x				x			x				
Rossen et al. [59]	95.5	x			x			x					x			x		x	
Rothman et al. [60]	81.8	x		?	?	?	?	x						x			x	x	
Salmon et al. [83]	95.5	x			x					x		x			x				
Steinbach et al. [78]	77.3	x					x		x				x				x		
Su et al. [61]	90.9	x					x	x				x					x		
Timperio et al. [84]	95.5	x				x				x		x			x	x			
Timperio et al. [85]	95.5	x				x				x		x			x	x	x		
Trapp et al. [86]	90.9	x					x			x			x		x	x	x		
Trapp et al. [87]	95.5	x				x				x			x		x	x	x		
Zhu et al. [62]	86.4	x					x	x				x			x				
Zhu & Lee [63]	86.4	x					x	x				x			x				
Total		61	4	3	15	18	26	35	17	11	2	40	26	1	32	19	32	11	1

**Table 2:** Relation between physical environment and active transportation

	Active transportation to school			Active transportation during leisure		
	Active transportation to school	Walking to school	Cycling to school	Walking/cycling during leisure	Walking during leisure	Cycling during leisure
WALKABILITY	3/5 +	4/5 +	2/3 ?	0/0 ?	0/2 ?	0/1 ?
Density	3/10 0	6/8 +	0/2 ?	0/0 ?	3/8 (+)	0/4 ?
Land use mix diversity	3/10 0	7/20 (+)	0/3 ?	0/0 ?	1/10 0	1/4 ?
Street connectivity	2/17 0	9/30 0	1/8 0	0/8 0	5/12 (+)	2/6 ?
ACCESSIBILITY	5/11 (+)	6/8 +	1/2 ?	0/0 ?	2/2 ?	0/0 ?
WALK/CYCLE FACILITIES	5/23 0	16/29 (+)	1/11 0	2/7 0	4/13 0	2/11 ?
AESTHETICS	3/17 0	4/23 0	1/6 0	0/3 ?	0/4 ?	0/2 ?
SAFETY	10/21 (+)	11/22 (+)	3/10 0	0/2 ?	0/0 ?	0/0 ?
Crime safety	1/10 0	5/16 0	0/1 ?	0/7 ?	2/4 ?	0/0 ?
Traffic safety	18/42 (+)	32/71 (+)	12/27 (+)	5/33 0	4/17 0	3/7 0
RECREATION FACILITIES	4/8 (+)	2/7 0	1/2 ?	3/7 (+)	4/11 (+)	1/6 0

n/N = number of positive associations/number of total investigated associations

0 = 0–33 % of the findings supporting the association = unrelated evidence

(+) or (−) = 34–59 % of the findings supporting the association = evidence for a possible association

+ or - = 60–100 % of the findings supporting a positive or negative association = convincing evidence

? =variable was investigated in less than three studies

**Table 3:** Relation between physical environment and active transportation across continents

	Active transportation to school					Active transportation during leisure				
	n/N relation Europe	n/N relation North-America	n/N relation Australia	n/N relation Asia	Total relation	n/N relation Europe	n/N relation North-America	n/N relation Australia	n/N relation Asia	Total relation
WALKABILITY	3/5 +	3/3 ?	3/5 +	0/0 ?	9/13 +	0/3 ?	0/0 ?	0/0 ?	0/0 ?	0/3 ?
Density	1/4 0	8/14 (+)	0/0 ?	0/1 ?	9/19 (+)	0/1 ?	1/4 ?	0/0 ?	2/8 ?	3/13 0
Land use mix diversity	1/10 0	9/22 (+)	0/0 ?	0/0 ?	10/32 0	0/0 ?	1/6 ?	0/0 ?	1/8 ?	2/14 0
Street connectivity	3/19 0	8/28 0	1/6 0	0/0 ?	12/53 0	1/8 ?	1/4 ?	0/6 ?	5/8 ?	7/26 0
ACCESSIBILITY	2/3 ?	6/12 (+)	3/6 (+)	0/0 ?	11/21 (+)	0/0 ?	2/2 ?	0/0 ?	0/0 ?	2/2 ?
WALK/CYCLE FACILITIES	5/23 0	15/31 (+)	1/8 0	0/0 ?	21/62 0	3/10 ?	1/2 ?	1/3 ?	3/16 ?	8/31 0
AESTHETICS	1/17 0	7/29 0	1/3 ?	0/0 ?	9/49 0	0/7 ?	0/2 ?	0/0 ?	0/0 ?	0/9 0
SAFETY	8/25 0	10/21 (+)	6/7 +	0/0 ?	24/53 (+)	0/2 ?	0/0 ?	0/0 ?	0/0 ?	0/2 ?
Crime safety	0/2 ?	6/18 0	0/8 0	0/0 ?	6/28 0	1/3 ?	1/2 ?	0/6 ?	0/0 ?	2/11 0
Traffic safety	10/31 0	24/54 (+)	28/55 (+)	0/0 ?	62/140 (+)	3/19 0	0/2 ?	3/27 0	5/8 ?	12/57 0
RECREATION FACILITIES	1/8 ?	4/7 (+)	2/2 ?	0/0 ?	7/17 (+)	1/8 0	4/4 ?	3/4 ?	0/8 ?	8/24 0

n/N = number of positive associations/number of total investigated associations

0 = 0–33 % of the findings supporting the association = unrelated evidence

(+) or (−) = 34–59 % of the findings supporting the association = evidence for a possible association

+ or - = 60–100 % of the findings supporting a positive or negative association = convincing evidence

? = variable was investigated in less than three studies

### General relationship between physical environment and walking and cycling

#### Active transportation to school

Table 2 summarizes the physical environmental characteristics that are investigated in relation to ATS in 57 studies. The complete table is presented in Additional file 2. Twenty-eight studies investigated this relationship separately for WTS and CTS [15, 23, 34, 39, 45, 46, 48, 50, 52, 54–64, 67, 70, 76–78, 86, 87, 89].

ATS was positively related to walkability (composed of three environmental attributes: residential density, intersection density and land use mix [90]). Evidence for a possible positive relationship with ATS was found for accessibility, general safety, traffic safety and recreation facilities. ATS was unrelated to density, land use mix diversity, street connectivity, walk/cycle facilities, aesthetics and crime safety. WTS was positively related to walkability, density and accessibility. Evidence for a possible positive relationship with WTS was found for land use mix diversity, safety and traffic safety and walk/cycle facilities. WTS was unrelated to street connectivity, aesthetics, crime safety and recreation facilities. Evidence of a possible positive relation with CTS was found for traffic safety. CTS was unrelated to street connectivity, walk/cycle facilities, aesthetics and safety.

39.7 % of the objective physical environmental characteristics were related to walking or cycling to school, and 39.1 % of the subjective physical environmental characteristics were related to walking or cycling to school.

#### Active transportation during leisure time

Twelve studies investigated environmental correlates of walking or cycling for transportation during leisure time in children. An overview of the results of these studies is given in Table 2. The complete table is presented in Additional file 2. Studies investigated walking and cycling separately [23, 38, 65, 69, 78, 88] or studied walking and cycling together [31, 71, 72, 79, 84] and one study investigated walking and cycling separately, as well as walking and cycling together [15].

Evidence for a possible positive association with walking or cycling for transportation during leisure was found for recreation facilities. Walking or cycling for transportation during leisure was unrelated to street connectivity, walk/cycle facilities and traffic safety. Evidence for a possible positive association with walking for transportation during leisure was found for density, street connectivity and recreation facilities. Walking was not associated with land use mix diversity, walk/cycle facilities and traffic safety. Cycling for transportation during leisure was not associated with traffic safety and recreation facilities.

23.0 % of the objective physical environmental characteristics were related to walking or cycling during leisure, and 24.5 % of the subjective physical environmental characteristics were associated with walking or cycling during leisure.

### Continent specific relationships between physical environment and PA

#### Active transportation to school

Table 3 provides a summary of the association between the physical environment and active transportation to school across different continents. The complete table is presented in Additional file 2. In Europe, a positive association was found between active transportation to school and walkability. Density, land use mix diversity, street connectivity, walk/cycle facilities, aesthetics, general safety and traffic safety were not associated with active transportation to school in Europe. In North America (USA and Canada), evidence for a possible positive association with active transportation to school was found for density, land use mix diversity, accessibility, walk/cycle facilities, general safety, traffic safety and recreation facilities. Active transportation to school was unrelated to street connectivity, crime safety and aesthetics. In Australia, positive associations of active transportation to school with walkability and general safety. Evidence for a possible positive association was found for accessibility and traffic safety. No association was found between active school transportation and street connectivity, walk/cycle facilities and crime safety. In Asia, none of the investigated physical environmental characteristics were sufficiently investigated in relation to active transportation to school to be able to draw relevant conclusions.

### Walking/cycling for transportation during leisure time

Table 3 provides a summary of the relation between physical environmental variables and walking and cycling for transportation during leisure time in children across different continents. The complete table is presented in Additional file 2. In Europe, no association was found between walking or cycling for transportation during leisure time and traffic safety and recreation facilities. In Australia, no association was found between walking or cycling for transportation during leisure time and traffic safety. All other physical environmental variables were insufficiently investigated in relation to walking and cycling for transportation during leisure time in the different continents to be able to draw conclusions.

## Discussion

This systematic review reported on the associations between a wide range of physical environmental characteristics and active transportation in 6- to 12-year-old-children across different continents. Based on the systematic review of 65 studies, evidence was found for different associations between the physical environment and active transportation in children.

In general, most significant associations between the physical environment and different contexts of active transportation were observed for active transportation to school. In particular, walkability was positively associated with active transportation to school in general (walking or cycling) and walking to school; indicating that children who were exposed to more walkable neighborhoods (high street connectivity, high land use mix diversity and a high residential density) were more likely to actively commute to school.

A possible positive association was found between traffic safety and all forms of active transportation to school. Furthermore, evidence for a possible positive relation was found between general safety and active transportation to school in general and walking to school. This may indicate that a neighborhood that is safe from traffic is an important condition for children to commute actively to school.

This review showed that walkability, density, land use mix diversity, accessibility, walk/cycle facilities and traffic safety were positively associated with different contexts of active transportation to school. So, a physical activity friendly neighborhood is related to more active transportation to school. Aesthetics was a largely investigated variable in relation to active transportation, but was unrelated to any form of active transportation, indicating that whether or not the neighborhood is aesthetically pleasing, is unimportant for children to commute actively to school. Similar to our results concerning active transportation to school, Ding et al. found positive associations between walkability, residential density and traffic safety and children’s overall physical activity [13].

The association between the physical environment and walking and cycling for transportation during leisure time was less clear. Evidence for possible positive associations was only found between recreation facilities and active transportation during leisure (walking/cycling and walking for transportation during leisure) and between street connectivity, density and walking for transportation during leisure. On one hand, this may be due to the fact that the physical environment was much less investigated in relation to active transportation during leisure (less than 20 % of the included studies) compared to active transportation to school. Hereby, there was insufficient evidence to make any conclusions on the association between different physical environmental factors and active transportation during leisure. On the other hand, this can be due to the fact that the prevalence of children who are actively commuting to school is much higher than children walking and cycling for transportation during leisure time [84] and that children who walk or cycle during leisure are mostly accompanied by a parent or a friend, making other influences (e.g. parental attitude or encouragement or friends living in the neighborhood) more important compared to the physical environment.

Across continents, associations between the physical environment and active transportation to school were mainly found in North America and Australia. In Europe a significant association was only found between walkability and active transportation to school. General safety and traffic safety were more frequently related to active transportation to school in North America and Australia compared to Europe. Also in adults, safety measures were unrelated to PA in Europe [8]. This might be due to the fact that walking and cycling to school are more dangerous in the USA and Australia compared to Europe [20]. In Europe, large efforts have been done to increase safety around schools. Therefore, in Europe active transportation may be less dependent on safety of the neighborhood as general safety is rather high. Other continent specific relations with active transportation to school were found for density and walk/cycle facilities; showing positive associations with density in North America, but not in Europe; and showing evidence for a possible positive association with walk/cycle facilities in North America, but not in Europe and Australia. This review indicated that there was more evidence for relations between density, land use mix diversity, accessibility and walk/cycle facilities in North America compared to other continents. The continent specific relationships in Europe, Australia and North America were probably due to differences in the physical environment in general, and in design of land use and traffic and crime situations in specific. Besides the differences in physical environments across the different continents, walking and cycling behaviors across continents differ. Cycling rates, for example, are higher in Europe, compared to cycling rates in the USA, Australia or Canada [21].

In Asia, the relationship between the physical environment and active transportation to school was insufficiently investigated to be able to draw relevant conclusions. As only two studies investigated the relationship between the physical environment and active transportation in Asia; and no studies conducted in Africa or South-America; there is a need for similar studies in Africa, Asia, and South-America. Most of the physical environmental variables were insufficiently investigated in relation to active transportation during leisure time to draw relevant continent-specific conclusions. It could only be concluded that traffic safety was not associated with active transportation during leisure in Europe and North America and that recreation facilities were not associated with active transportation during leisure time in Europe. Therefore, future studies should specifically focus on active transportation during leisure.

As the perception of the neighborhood can differ from the objectively determined neighborhood, it is expected that study outcomes differ according to the measurement method of the environment. In adults and adolescents it was shown that neighborhood perception was more strongly related to PA compared to the objectively determined neighborhood [91, 92]. On the contrary, Ding et al. found in their review more consistent associations with PA in children, when the objectively determined neighborhood was involved [13]. According to our results, the objective and subjective neighborhood characteristics were equally related to different contexts of active transportation compared to objective characteristics. Therefore, in future research, it is advisable to determine the association between objective as well as subjective neighborhood characteristics in relation to children’s active transportation. Furthermore, there is a need for clearly defined concepts (e.g. the walkability index) and univocal instruments to determine the environment, as in the included studies, different measurement methods were used to measure the same environmental characteristics. This makes it more difficult to compare the different results and to draw univocal conclusions.

Only four studies out of 65, used a longitudinal design. Only studies with a longitudinal design can investigate causal interference and have the potential to evaluate which environmental factors predict positive versus negative changes in the level of active transportation and how environmental perceptions change as children grow up. Also studies including social and psychological factors are necessary, as it has been shown in previous studies [29, 76] that these factors are important correlates of children’s active transportation. Furthermore, some studies included in this study indicated that associations between the physical environment and children’s active transportation may vary according to different subgroups (e.g. high vs. low SES). Therefore, in future research, there is also a need for studies across different subgroups, to identify groups in need of targeted interventions. As this review study indicates that continent-specific study findings are not generalizable to other continents, studies from Africa, Asia and South-America are necessary.

This review is the first to provide a wide overview of all physical environmental correlates of specific contexts of active transportation in children. A strength of this study is the specific age-range (6–12 year) in which these correlates were investigated and the fact that different contexts of active transportation were studied separately and across different continents. A limitation of this review is that only the physical environmental correlates were studied. It is highly probable that children’s walking and cycling levels are associated to other factors, such as socioeconomic status, weather, behavioral constructs, cultural factors, distance to school and walk/bike to school groups. Furthermore, it is possible that other factors that differ across continents (e.g. cultural or social factors) may have confounded the findings concerning the association between active transportation and the physical environment across continents. Besides, it is likely that the association between the physical environment and children’s active transportation also differs within continents and that some results are country specific. Another issue that needs to be taken into account is the possible presence of a publication bias. As investigators may be inclined to publish statistically significant results rather than results in which no associations were found, there might be an underrepresentation of studies where no significant relations were found [93] and this could have led to type 1 errors in the current review. It is also expected that researchers are less inclined to publish results in the opposite direction of their hypothesis. Prevention of publication bias may be done by encouraging researchers to publish non-significant results and by registering every trial undertaken, however, this is an ideal that is hard to achieve [94]. Also the focus on English-written articles only forms a limitation of this study. The lack of Asian, South-American and Asian studies can be attributable to this limitation. A last limitation is the use of a vote count review method, which gives the same weight to results from different studies, independent of the sample size, design and quality of the studies. It is possible that conclusions of this review may be influenced by studies with small sample sizes or by studies that reported many results. As there was large heterogeneity in the included studies concerning the methods of the physical environment measurement, type of physical activity measure that was used and the use of adjusting variables, the data were not meta-analyzed.

## Conclusions

An activity friendly neighborhood that is walkable, dense, accessible, equipped with walk/cycle facilities and safe from traffic is associated with more active transportation to school in children. Only a limited number of studies assessed the association between the physical environment and children’s walking or cycling for transportation during leisure, which limited the ability to draw conclusions on this association. Aesthetics were not associated with any context of active transportation. Some continent specific relations were found. Safety measures were more important in relation to active commuting to school in North America and Australia compared to Europe. There is a need for longitudinal studies, and studies conducted in Asia, Africa and South-America.

## Additional files

Additional file 1:
**Overview of the characteristics and results of the included studies.** (DOCX 218 kb) Additional file 2:
**Complete overview of the associations between the physical environment and physical activity.** (DOCX 58 kb)

## Abbreviations

ATS: active transportation to school
CTS: cycling to school
GIS: geographical information system
WTS: walking to school
