# Supplementary material for: Cross-continental comparison of the association between the physical environment and active transportation in children: a systematic review
Source: Int J Behav Nutr Phys Act. 2015 Nov 26;12:145. doi: 10.1186/s12966-015-0308-z (PMC4660808; doi:10.1186/s12966-015-0308-z)
Supplement: Additional file 1: — Overview of the characteristics and results of the included studies. (DOCX 218 kb) [file 12966_2015_308_MOESM1_ESM.docx]

| Study | Sample size/ age/Country | Design | Environmental measurement | PA measurement | Associations | Features |
| --- | --- | --- | --- | --- | --- | --- |
| (Aarts et al. 2013) | n=5963 from 44 primary schools,  4-12 yr (mean age 7.8 ±2.4 year),  The Netherlands | CS | Parental questionnaire: perception of the neighborhood environment | *Parental questionnaire*  Walking to school (usual transport mode) | *Multilevel multinomial logistic regression analyses*  AESTHETICS  0 Presence of trash and litter  - Presence of green  <> AESTHETICS  + Presence of dog dirt  <>TRAFFIC SAFETY  0 Traffic situation  WALK/CYCLE FACILITIES  0 Quality of sidewalks and bike lanes | *RR schools= 36.5%  *RR parents = 60%  * traffic situation: higher scores represent less favorable traffic situation  * controlled for age, distance from home to school and parental education  * traffic situation: higher scores represent less favorable traffic situation |
|  |  |  |  | Cycling to school (usual transport mode) | *Multilevel multinomial logistic regression analyses*  AESTHETICS  0 Presence of trash and litter  0 Presence of green  <> AESTHETICS  0 Presence of dog dirt  <>TRAFFIC SAFETY  - Traffic situation  WALK/CYCLE FACILITIES  0 Quality of sidewalks and bike lanes |  |
| (Alton et al. 2007) | n=473 from 6 elementary schools,  9-11 yr  UK: Birmingham | CS | children’s and parental questionnaire: perception of the neighborhood environment | *Children’s questionnaire*  high levels of walking (children who walked more than the mean number of walking trips were classified as high walkers) | ***Binary logistic regression***  *Parental perception:*  <>TRAFFIC SAFETY  **+ heavy traffic in the street where  you** **live**  **+ anxiety about safety of roads**  <>CRIME SAFETY  0 parental warning about strangers   when out alone  *Children’s perception:*  <>TRAFFIC SAFETY  **+ anxiety about heavy traffic in the  street where you live**  **+ anxiety about safety of roads**  <> CRIME SAFETY  - worry about strangers when out  alone  RECREATION FACILITIES  0 no parks or grounds near where you  live | * RR= 82%  * purposive sampling: one school was of high, three of medium and two of low socioeconomic classification.  * school was not included as a destination in the questionnaire  * Binary logistic regression is adjusted for: school year, sex, ethnicity, car ownership, number of rooms in principal house |
| (Braza et al. 2004) | n=2993  9-11 yr  USA: California | CS | GIS: school environment | hand counts in 5^th^ grade classrooms of biking/cycling rates  ATS =  students were asked to raise their hands to indicate how they arrived on school that morning (walking and biking rates) | *Pearson correlation*  RESIDENTIAL DENSITY  **+ residential population within 0.5  mile of school**  STREET CONNECTIVITY  + number of intersections per street   mile within 0.5 mile of school  ***Multiple regression model*** | * school that participated in the October 1999 Walk to School Day  * RR schools = 20%  * RR=23.3%  * neighborhood within 0.5 mile from school  * Multiple regression model is controlled for total school enrollment and school demographic characteristics  * significance was set at p<0.10  * bold: significant in multiple regression |
| (Bringolf-Isler et al. 2008) | n=1031  6-14 yr (M=10.95±3.25 yr)  Switzerland: Payerne – Bern – Biel/Bienne | CS | GIS (route to school and neighborhood)  parental perception of the route to school | Parental questionnaire  ATS=  walking or using a bike/kick scooter/inline skates as main mode of transportation to school  AV = non active commuting as main mode of transportation | *Multivariate logistic regression analysis*  parental perception:  SAFETY  0 safety of the way to school  GIS:  TRAFFIC SAFETY  0 motorway crossings on the way to  school  RESIDENTIAL DENSITY  0 population density around home | * RR 65 %  * controlled for: age group, community (Bern, Biel, Payerne), number of cars  * SCARPOL study  * main street crossing = busy road barrier |
| (Carson et al. 2010) | n=3028  Grade 5 students  Canada: Alberta | CS | Parental perception of the neighborhood environment | *Parental questionnaire*  ATS=  transport was categorized as “active” if the child usually walked or biked to and from school | *Multilevel logistic regression analysis*  WALK/CYCLE FACILITIES  RECREATION FACILITIES  + sidewalks / parks  ACCES  0 neighborhood satisfaction / services  GENERAL SAFETY  0 safety | * Raising healthy Eating and Active Living Kids in Alberta (REAL Kids Alberta)  * RR: schools 80.4%  * RR students 61.2%  * adjusted for: gender, geographic region, household income, parental education  * safety = It is safe for children to play outside during play. / Traffic makes my neighborhood an unsafe place for my child. / Crime makes my neighborhood an unsafe place for my child.  * sidewalks/parks = In my neighborhood there are good parks, playgrounds, and/or places to play./ In my neighborhood there are sidewalks on most of the streets  * neighborhood satisfaction/services = I like where I live. / My grade five child has good access to sports and recreation. / I have good access to stores to purchase fresh fruits and vegetables. |
| (Carver et al. 2008) | n=188  8-9yr (M=9.1±0.4yr)  Australia: Melbourne | CS | GIS  (environment around home, radius 800m) | Parental report  Habitual walking/cycling=  How frequently their child usually walked/cycled to 15 specific destinations (dichotomized: less than 7 trips/weeks or more than 7 trips per week) | *Bivariate logistic regression analyses*  STREET CONNECTIVITY  0 total number of intersections  0 residing on a cul-de-sac  WALK/CYCLE FACILITIES  0 length of walking tracks  TRAFFIC SAFETY  0 number of speed humps  0 gates/barriers  0 number of traffic lights  0 slow points  ***Multiple logistic regression*** | * Children living in active neighborhoods study (CLAN)  * RR: 27% at baseline, 76% FU  * results from the 3 year follow up  * Total length of local roads: total length (km) of roads classed as “local”, “two-wheel drive”, or “four-wheel drive” within the State Government of Victoria’s road hierarchy[26](http://www.ncbi.nlm.nih.gov/pmc/articles/PMC2443253/#CR26) was computed. These roads generally have a maximum speed limit of 50 km/h;[21](http://www.ncbi.nlm.nih.gov/pmc/articles/PMC2443253/#CR21)  * Local road index ratio: ratio of total length of local roads to total length of all roads, i.e., the ratio of roads with lower speed limits/traffic volume to all roads  * Intersection density: usually refers to the number of intersections per unit of area, but neighborhoods in this study had identical area, so number of intersections per neighborhood was entered into analyses.  * Residing on a cul-de-sac: indicates whether or not a participant resided on a cul-de-sac or no-through road (defined in GIS as a road segment that contained a road end).  * Total length of walking tracks: total length (m) of walking tracks identified from road network data  *gates/barriers en road: may have a traffic-calming effect |
| (Carver et al. 2010) | n=177  8-9 yr => 10-11yr  Australia: Melbourne | L | GIS within each participant’s neighborhood | *Parental report*  change in number of walking/cycling trips made to 15 specific destinations | *Bivariate linear regression analysis*  STREET CONNECTIVITY  0 intersection density  0 residing on a cul-de-sac  WALK/CYCLE FACILITIES  + length of walking tracks^g^  TRAFFIC SAFETY  0 number of speed humps  + number of traffic/pedestrian lights^g^  0 gates/barriers  0 slow points  ***Multiple regression analysis*** | * Children living in active neighborhoods (CLAN)  * results from the 3 year follow up  * Total length of local roads: total length (km) of roads classed as “local”, “two-wheel drive”, or “four-wheel drive” within the State Government of Victoria’s road hierarchy[26](http://www.ncbi.nlm.nih.gov/pmc/articles/PMC2443253/#CR26) was computed. These roads generally have a maximum speed limit of 50 km/h;[21](http://www.ncbi.nlm.nih.gov/pmc/articles/PMC2443253/#CR21)  * Local road index ratio: ratio of total length of local roads to total length of all roads, i.e., the ratio of roads with lower speed limits/traffic volume to all roads  * Intersection density: usually refers to the number of intersections per unit of area, but neighborhoods in this study had identical area, so number of intersections per neighborhood was entered into analyses.  * Residing on a cul-de-sac: indicates whether or not a participant resided on a cul-de-sac or no-through road (defined in GIS as a road segment that contained a road end).  * Total length of walking tracks: total length (m) of walking tracks identified from road network data |
| (Chillon et al. 2014) | N=1007  4^th^-5^th^ grade  USA | CS | Parental survey about barriers (neighborhood)  Audit within 1 mile from school | *Children’s questionnaire*  ATS (total number of active trips) | *Bivariate generalized linear mixed model with log link functions*  WALK/CYCLE FACILITIES  - walkability/bikability school environment (audit)  + suitability of the route to school (parental report)  SAFETY  + safety and weather (parental report)  (higher scores denote better conditions) | * National Evaluation of Walk to School Project  * external safety and weather = bullies, kidnapping, arriving safely to school, weather, unleashed dogs and traffic congestion  * suitability of the route = lack of sidewalks and crosswalks, steep hills, areas without people around, speed and traffic and insufficient daylight in the morning  * audit instrument: The Walkability and Bikeability Suitability Assessment (WABSA) protocol; higher score indicating more hazardous environment  * walkability suitability= vehicle traffic and speed, pedestrian signs, suitability of sidewalks  * bikeability suitability = vehicle traffic and speed and suitability of roads (e.g. bike lane presence, width etc.) |
| (Christiansen et al. 2014) | N=1250  11-13 year  Denmark | CS | GIS  Children’s questionnaire | *5 day active commuting diary filled out by children*  ATS | *Bivariate logistic regression analyses*  WALKABILITY  **+ school walkability (GIS)**  SAFETY  **+ safety of the route to school**  WALK/CYCLE FACILITIES  **+ cycle paths in area where I live**  TRAFFIC SAFETY  + safe crossings in area where I live  <>TRAFFIC SAFETY  - heavy traffic in area where I live  **- cars drive fast in area where I live**  ***Multivariable logistic regression analysis*** | * 14 schools  * baseline measurements of the SPACE-for physical activity study  * RR=92.7%  * school walkability = road connectivity, vehicular traffic exposure, residential density  * safety of the route to school: “how would you describe your cycle route to or from school?” |
| (Curriero et al. 2013) | N= 362, from 6 schools  8-12 years,  Baltimore, USA | CS | GIS  Audit data  Children’s and parental perception | *Child questionnaire*  WTS | *Univariate logistic regression*  SAFETY  0 child’s perception of neighborhood safety  **+ parental perception of neighborhood safety**  0 children’s perceived safety of the neighborhood on the way to school  WALK/CYLE FACILITIES  **- walking path length to school (audit + GIS)**  + walking path quality to school (audit + GIS)  <>AESTHETICS  **+ incivility for home street block (audit + GIS)**  ***Multilevel, multivariate GEE*** | * Neighborhood Inventory for Environmental Typology (NIfETy) = street block level auditing instrument  * RR schools = 75%  * incivility = calculated by summing 49 items from the VAOD (violence, alcohol and other drugs), physical disorder (e.g. broken windows) and social disorder (e.g. people fighting, evidence of prostitution) |
| (Cutumisu et al. 2013) | N=809  9-13 yr, Quebec Canada | CS | Parental and children’s perceptions of their neighborhood | *Parental questionnaire*  ATS (how many days/week does your child walk/bike to school? (active: 3 or more days per week) | *Logistic regression analysis*  SAFETY  0 child’s perceived safety  + parental perceived safety  ***Multivariate logistic regression analysis*** | * Baseline data from a larger study using a repeated cross-sectional design over years to evaluate the effects of the Operation Wixx multimedia campaign  * children’s perceived neighborhood safety: “I feel safe to engage in physical activity in my neighborhood”  * parental perceived safety: “I feel at ease to let my child travel on foot or on bicycle from home to school” |
| (de Vries et al. 2010) | n=448  6-11 yr old  (M=8.3±1.5 year)  The Netherlands | CS | NEWS completed by specialists after observation of the neighborhood | *7 day physical activity diary completed by children + parent*  Walking and cycling for transportation | *Multilevel univariate analysis*  RECREATION FACILITIES  0 Play facilities  0 recreation facilities  AESTHETICS  0 green space  0 proportion green space to residents  TRAFFIC SAFETY  0 traffic safety  0 traffic lights  0 traffic islands  WALK/CYCLE FACILITIES  **+ sidewalks**  0 cycle-tracks  0 pedestrian crossings  STREET CONNECTIVITY  0 roundabouts  0 intersections  ***Multilevel multivariate analysis*** | * RR 51%  * adjusted for: age, sex, parental education level and ethnicity and significant variables in model  * SPACE-study: spatial planning and children’s exercise |
|  |  |  |  | *7 day physical activity diary completed by children + parent*  Walking for transportation | *Multilevel univariate analysis*  RECREATION FACILITIES  0 Play facilities  0 recreation facilities  AESTHETICS  0 green space  0 proportion green space to residents  TRAFFIC SAFETY  0 traffic safety  **- traffic lights**  0 traffic islands  WALK/CYCLE FACILITIES  0 sidewalks  + cycle-tracks  0 pedestrian crossings  STREET CONNECTIVITY  **+ roundabouts**  0 intersections  ***Multilevel multivariate analysis*** |  |
|  |  |  |  | *7 day physical activity diary completed by children + parent*  Cycling for transportation | *Multilevel univariate analysis*  RECREATION FACILITIES  0 Play facilities  **+ recreation facilities**  AESTHETICS  0 green space  0 proportion green space to residents  TRAFFIC SAFETY  + traffic safety  - traffic lights  0 traffic islands  WALK/CYCLE FACILITIES  - sidewalks  0 cycle-tracks  **+ pedestrian crossings**  STREET CONNECTIVITY  0 roundabouts  0 intersections  ***Multilevel multivariate analysis*** |  |
|  |  |  |  | *7 day physical activity diary completed by children + parent*  Walking and cycling to school | *Multilevel univariate analysis*  RECREATION FACILITIES  0 Play facilities  0 recreation facilities  AESTHETICS  0 green space  0 proportion green space to residents  TRAFFIC SAFETY  0 traffic safety  0 traffic lights  0 traffic islands  WALK/CYCLE FACILITIES  0 sidewalks  **+ cycle-tracks**  0 pedestrian crossings  STREET CONNECTIVITY  0 roundabouts  0 intersections  ***Multilevel multivariate analysis*** |  |
|  |  |  |  | *7 day physical activity diary completed by children + parent*  Walking to school | *Multilevel univariate analysis*  RECREATION FACILITIES  0 Play facilities  0 recreation facilities  AESTHETICS  **- green space**  0 proportion green space to residents  TRAFFIC SAFETY  0 traffic safety  0 traffic lights  0 traffic islands  WALK/CYCLE FACILITIES  0 sidewalks  0 cycle-tracks  **+ pedestrian crossings**  STREET CONNECTIVITY  **+ roundabouts**  0 intersections  ***Multilevel multivariate analysis*** |  |
|  |  |  |  | *7 day physical activity diary completed by children + parent*  Cycling to school | *Multilevel univariate analysis*  RECREATION FACILITIES  0 Play facilities  **+ recreation facilities**  AESTHETICS  + green space  0 proportion green space to residents  TRAFFIC SAFETY  0 traffic safety  - traffic lights  0 traffic islands  WALK/CYCLE FACILITIES  0 sidewalks  0 cycle-tracks  **+ pedestrian crossings**  STREET CONNECTIVITY  0 roundabouts  0 intersections  ***Multilevel multivariate analysis*** |  |
| (Deweese et al. 2013) | n=901  3-18 (mean age 10.26 ± 4.08 years),  6-11 years,  New Jersey, USA | CS | Parental questionnaire: perception of their neighborhood | *Parental questionnaire*  Engaging in ATS ≥ 1 day per week | *Bivariate logistic analyses*  TRAFFIC SAFETY  0 traffic safety  CRIME SAFETY  0 crime safety  <>AESTHETICS  - unpleasantness of walking  WALK/CYCLE FACILITIES  0 sidewalk condition | * RR=49%  * unpleasantness of walking = ‘how pleasant is it to walk, run, bike, or play in your neighborhood? For example, are there trees and proper lighting, no graffiti or abandoned buildings? |
| (D'Haese et al. 2011) | n=696  10-11 yr (M=11.2±0.5)  Europe, Belgium | CS | parental NEWS | *parental questionnaire*  ATS | *Multivariate logistic regression analyses*  WALKABILITY  0 walkability  ACCESSIBILITY  + accessibility  WALK/CYCLE FACILITIES  0 walk/cycle facilities  AESTHETICS  0 neighborhood aesthetics  TRAFFIC SAFETY  0 safety from traffic  CRIME SAFETY  0 safety from crime | * RR schools=42.9%  RR parents=69.9%  * controlled for SES, gender, number of motorized vehicles |
| (D'Haese et al. 2014) | N=474  18 schools,  9-12 year  Ghent, Belgium | CS | GIS | *Parental questionnaire*  ATS | *Linear regression analyses*  WALKABILITY  0 walkability | * Belgian Environmental and Physical Activity Study in children (BEPAS-child study)  *RR schools=34.6%  *RR parents=61.0%  * controlled for age, sex, family SES |
|  |  |  |  | *Parental questionnaire*  Walking for transportation during leisure | *Linear regression analyses*  WALKABILITY  - walkability (only in low SES neighborhoods)  0 walkability (in high SES neighborhoods) |  |
|  |  |  |  | *Parental questionnaire*  Cycling for transportation during leisure | *Linear regression analyses*  WALKABILITY  0 walkability |  |
| (Ducheyne et al. 2012) | N=850  10-12yr (M=10.38±0.95)  Belgium | CS | Parental NEWS: neighborhood environment | *Parental questionnaire*  CTS (always cycling to school) | *Bivariate logistic regression*  RESIDENTIAL DENSITY  0 residential density  WALK/CYCLE FACILITIES  0 walking and cycling facilities  STREET CONNECTIVITY  0 connectivity  AESTHETICS  0 aesthetics  TRAFFIC SAFETY  **+ traffic safety**  CRIME SAFETY  0 crime safety  ***Multivariate model*** | * RR schools = 44%  * RR parents = 80%  * controlled for age, gender, BMI, household car access |
| (Durand et al. 2012) | N=365  11.7 year  USA, California | CS | NEWS completed by parents | *Self-report by children*  ATS (=usual transportation mode to school (active vs. passive)) | *Multivariate logistic regression*  RESIDENTIAL DENSITY  0 residential density  LAND USE MIX DIVERSITY  - Land use mix diversity  LAND USE MIX ACESSIBILITY  0 land use mix access  0 hilliness  0 physical barriers  CONNECTIVITY  0 lack of cul-de-sacs  0 street connectivity  0 cul-de-sac connectivity  <>CRIME SAFETY  0 crime  <>TRAFFIC SAFETY  0 traffic hazards  AESTHETICS  0 aesthetics  WALK/CYCLE FACILITIES  0 walking infrastructure | * RR=91%  * analyses controlled for child age, gender, race/ethnicity, free/reduced lunch status, community of residence dummy variable, and proximity to school |
| (Frank et al. 2007) | n=847  5-8 yr  USA: Atlanta | CS | GIS  Neighborhood environment (1km buffer) | *Strategies for Metropolitan Atlanta’s Regional Transportation and Air Quality (SMARTRAQ) household travel survey filled out by a legal guardian*  walked at least once over 2 days | *Logistic regression analyses*  STREET CONNECTIVITY  0 street connectivity  RESIDENTIAL DENSITY  0 residential density  LAND USE MIX DIVERSITY  0 mixed land use  0 at least one commercial land use  RECREATION FACILITIES  + at least one recreational/open space land use (vs. none) | recruitment rate = 44.8%  data retrieval success rate = 67.8%  Participation rate = 30.4% |
|  | n=632  9-11 yr  USA: Atlanta | CS | GIS  Neighborhood environment | *Stratetgies for Metropolitan Atlanta’s Regional Transportation and Air Quality (SMARTRAQ) household travel survey filled out by a legal guardian*  walked at least once over 2 days | *Logistic regression analyses*  STREET CONNECTIVITY  0 street connectivity  RESIDENTIAL DENSITY  + density  LAND USE MIX DIVERSITY  0 mixed land use  0 at least one commercial land use  RECREATION FACILITIES  + at least one recreational/open space land use |  |
| (Gallimore et al. 2011) | N=?  5 th grade  USA | CS | Irvine-Minnesota Inventory audit tool: route to school | *Parental or children’s questionnaire?*  WTS | *ANOVA*  TRAFFIC SAFETY  + traffic safety  ACCESSIBILITY  + accessibility  AESTHETICS  + pleasureability  CRIME SAFETY  + crime safety  RESIDENTIAL DENSITY  - density  LAND USE MIX DIVERSITY  0 diversity | *Traffic safety*: traffic features that facilitate or discourage pedestrian travel, e.g., stop lights, crosswalks, and speed limit signs  *Accessibility*: features that ease or impede walking from one area to another, e.g., sidewalks, ditches, and pedestrian access points in cul-de-sacs  *Pleasurability*: the pleasantness or unpleasantness of a pedestrian environment, e.g., street trees, overhead electric wires, and prominent garages  *Crime safety*: features related to crime safety or fear, e.g., bars on windows, outdoor lighting, and abandoned buildings  *Density of housing*: the type and size of buildings, e.g., single family detached homes, apartment buildings, and building height in stories  *Diverse destinations*: a list of destinations that reflect land use diversity, e.g., religious buildings, green spaces, and schools |
| (Giles-Corti et al. 2011) | n=1132 11.01±0.808 yr  Australia: Perth | CS | GIS: school neighborhood | *parental report*  regular WTS:≥6 trips/week | *logistic univariate model*  WALKABILITY  + school walkability index (within 2 km around school)  This effect attenuated after adjustment for clustering and distance to school | * RR schools: 69.4%  RR children: 56.6%  * Multivariate model = adjusted for clustering and individual-level demographic characteristics, SES of school,  * high walkable = high street connectivity (Pedshed) and low traffic volume |
| (He 2011) | n=3603 trips  5-12 yr  USA: Los Angeles, Orange, Riverside, San Bernardino, Ventura | CS | census data: school environment | *travel diary from Southern California Association of governments*  ATS | *multinomial logit model*  RESIDENTIAL DENSITY  0 school residential density | * impact of school locations |
| (Hsu and Saphores 2014) | N=729,  5-15 yr (mean age: 9.92 yrs), California USA | CS | Parental questionnaire: route to school and neighborhood | *Travel diary: California National Household Travel Survey*  ATS to school(vs. car, on the day of the survey to school) | *Multinomial logit model*  <>TRAFFIC SAFETY  - Concerns on traffic volume along route  0 Concerns on traffic speed along route  <>CRIME SAFETY  0 Concerns on crime/violence along route  RESIDENTIAL DENSITY  0 Housing density  0 Population density | * Only children included living less than 2 miles from school |
| (Hume et al. 2009) | n=121  9.1±0.34yr  (FU: age=11.1±0.33yr)  Australia: Melbourne | L | parental survey in neighborhood | *parental survey*  increase in ATS | *bivariable logistic regression analyses*  ACCESSIBILITY  0 hilly streets  STREET CONNECTIVITY  0 many cul-de-sacs, courts, or not-through roads near where I live  0 many alternative routes for getting from place to place  AESTHETICS  0 neighborhood is free from litter  <>TRAFFIC SAFETY  0 no traffic lights/crossings  0 heavy traffic in local streets  TRAFFIC SAFETY  0 road safety  CRIME SAFETY  0 stranger danger is a concern  WALK/CYCLE FACILITIES  0 satisfaction with number of pedestrian crossings in neighborhood  0 footpaths on most streets | * RR: 27% initial recruitement  * CLAN study: Children Living in Active Neighborhoods |
| (Johansson 2006) | N=248,  8-11 year old (mean age: 9.6yr),  Sweden | CS | Audit  Parental perceptions (trust in road users, environmental trust and trust in strangers) | *Parental travel diary*  WC (=journeys made alone or with friends to organized leisure activities) | *Multiple regression analyses*  TRAFFIC SAFETY  + traffic environment  0 trust in road users  WALK/CYCLE FACILITIES  0 footpaths and cycle paths  SAFETY  0 Environmental trust  CRIME SAFETY  0 trust in strangers | * AUDIT: traffic environment:  -there are are few busy roads  -cars are not parked closely along the streets  -noise and pollution from traffic are hardly noticed  -there are speed bumps or the like to limit the speed  -busy roads have crossings with traffic lights, there are foot-bridges or underpasses by busy roads  *footpaths and cycle paths  -the neighbourhood is well provided with footpaths and cycle paths  -the pavements are wide and well kept  -the street-lighting is sufficient  -pavements are properly separated from cycle lanes  -there are cycle lanes, which are properly separated from the traffic  -walkways and cycle paths are not surrounded by high bushes  -walkways and cycle paths are not perceived as dark and deserted  -underpasses feel light and open  *PERCEPTION PARENTS: trust in strangers:  - there is a risk that people may steal from my child  - there may be gangs of youth that harass my child  - my child may easily be offered drugs  - other children may threaten or harm my child  * PERCEPTION PARENTS: trust in road users  - I can trust that drivers notice my child  - professional drivers drive carefully when close by my child  - I can trust that my child is given right of way at pedestrian crossings  - cyclists take due consideration of my child  * PERCEPTIONS PARENTS: trust in physical environment:  - If my child plays outside I feel more safe if he/she stays close to our home  - I find it uncomfortable if my child has to walk along alleyways  - I think my child should be accompanied by an adult when it is dark outside  - I don’t feel comfortable letting my child walk along narrow pavements |
| (Kemperman and Timmermans 2014) | N=4293  4-11 years  The Netherlands | CS | Census data and survey from national representative sample | *Travel diary for children (completed with parental assistance if necessary)*  WC | *Bayesian belief network*  RECREATION FACILITIES  0 recreation areas  AESTHETICS  0 forests and nature areas  SAFETY  0 safety | * Only direct relationships were described  * safety = noise pollution, trouble caused by younger persons, and trouble caused by people living in the neighborhood |
| (Kerr et al. 2006) | N=259  5-18 yr (mean age: 11.3 yr)  USA: Seattle | CS | Parental questionnaire  NEWS: neighborhood | *Parental questionnaire*  ATS at least once a week | *Logistic regression analyses*  RESIDENTIAL DENSITY  0 residential density  LAND USE MIX DIVERSITY  0 land use mix diversity  + number of stores within a 20’ walk from home  ACCESSIBILITY  + land use mix acces  STREET CONNECTIVITY  + street connectivity  WC FACILITIES  + walk/cycle facilities  CRIME SAFETY  0 crime safety  TRAFFIC SAFETY  0 pedestrian traffic safety  AESTHETICS  + aesthetics | * Neighborhood Quality of Life Study (NQLS)  * RR=28% (adult study)  * RR=93% (re-contact adults for child study)  * logistic regression analyses controlled for: age, gender and parent education |
|  |  |  | Geographical Information System (GIS) walkability: neighborhood | *Parental questionnaire*  ATS at least once a week | *Logistic regression analyses*  LAND USE MIX DIVERSITY  0 mixed land use  STREET CONNECTIVITY  0 intersection density  RESIDENTIAL DENSITY  + Residential density  WALKABILITY  + individual walkability  + neighborhood walkability |  |
| (Kytta et al. 2012) | N=946  10-12 yr  Finland | CS | GIS (neighborhood) | *Children’s questionnaire*  ATS | *Logistic regression analyses*  RESIDENTIAL DENSITY  + residential density  AESTHETICS  - Proportion green space | * RR = 73%  * analysis adjusted for age and gender  * ATS = both journeys to and from school made actively (1) vs. 1 or both journeys inactively (0). |
| (Larouche et al. 2013) | N=29  Grade 6 | CS | Parental and children’s questionnaire | *Children’s travel diary*  ATS (active travel for at least 50% of the school trips) | *Fisher’s exact test*  Children’s perception  <>LAND USE MIX ACCESSIBILITY  0 Too many hills along the way  <>WALK/CYCLE FACILITIES  0 No sidewalks or bike lanes  <>AESTHETICS  0 Route is boring  <> TRAFFIC SAFETY  - Route doesn’t have good lighting  0 Too much traffic along the route  0 Too much traffic around home  - Too much traffic around the school  0 Dangerous crossings  Parental perception  <>LAND USE MIX ACCESSIBILITY  0 Too many hills along the way  <>WALK/CYCLE FACILITIES  0 No sidewalks or bike lanes  <>AESTHETICS  0 Route is boring  <> TRAFFIC SAFETY  - Route doesn’t have good lighting  - Too much traffic along the route  - Too much traffic around home  0 Too much traffic around the school  0 Dangerous crossings | * RR=33.3% |
| (Larouche et al. 2014) | N=567, mean age 10.0 years (grade 5),  Ottawa, Canada | CS | School’s administrator survey  School audit | *Children’s questionnaire*  *ATS (mean part of journey to school: active vs. inactive)* | *Generalized linear models*  School environment perceived by school administrator:  TRAFFIC SAFETY  + identify safe routes to school  **+ provides crossing guards**  0 designate car free zone  <> TRAFFIC SAFETY  0 traffic perceived as problem  <>AESTHETICS  0 garbage/litter perceived as problem  0 drugs/drinking perceived as problem  0 gangs perceived as problem  0 vacant/shabby housing perceived as problem  <>CRIME SAFETY  **+ crime perceived as problem**  School audit (school environment):  LAND USE MIX DIVERSITY  0 land use  WALK/CYCLE FACILITIES  0 cycle lane on the road  0 sidewalks on both sides  0 sidewalks on 1 side only  0 marked pedestrian crossing  0 route sign for cyclists  TRAFFIC SAFETY  + traffic calming measures  0 school warning sign for road users  0 road safety sign  **Multivariate model** | * International Study of Childhood Obesity, Lifestyle and the Environment (ISCOLE)  * RR schools = 50%  * models adjusted for school clustering |
| (Larsen et al. 2013) | N=905  Grade 5-6 (mean age 10.5yr)  Toronto, Canada | CS | Parental survey  Children’s survey  GIS  Observation (traffic density)  Route characteristics | *Parental survey*  WTS (vs. automobile traffic) | *Univariate logistic regression*  *To school*  *GIS (route to school characteristics)*  CONNECTIVITY  **- Intersections**  <> WALK/CYCLE FACILITIES  **- Proportion missing sidewalks**  <>TRAFFIC SAFETY  **- Maximum traffic**  TRAFFIC SAFETY  - Traffic-calming feature density  LAND USE MIX DIVERSITY  + Land use mix  <> TRAFFIC SAFETY  - Traffic density  AESTHETICS  + Street tree density  *Parental survey*  <> WALK/CYCLE FACILITIES  - Not enough sidewalks  TRAFFIC SAFETY  - Enough crosswalks  <> CRIME SAFETY  **- Fear of strangers**  <>TRAFFIC SAFETY  - Traffic around school  **- Crosses busy streets**  - Drivers are too fast  TRAFFIC SAFETY  - Crossing guards  SAFETY  0 Live in a safe area  *Children’s survey*  SAFETY  **+ Safe area to walk**  TRAFFIC SAFETY  0 Safe crossing roads  <> TRAFFIC SAFETY  0 Lots of traffic  0 Fast cars  <> CRIME SAFETY  0 Fear of strangers  0 Fear of older kids  **Multivariate binomial logistic regression** | * vehicle fleet index: fleet mix scores ranged from 0 to 1, where 0 is a mix dominated by trucks, transit, and school buses and a score of 1 represents an intersection with only passenger vehicles |
| (Lee et al. 2013) | N=1202 (601 child pairs), mean grade = 1.76, Austin, USA | CS | Parental survey | *Parental survey*  WTS (walkers vs. drivers) | *Paired sample t-tests/McNemar’s test*  RECREATION FACILITIES  0 Presence of a playground on home-to-school route  **+ presence of a park on home-to-school route**  WALK/CYCLE FACILITIES  0 presence of a walking path on home-to-school route  LAND USE MIX DIVERSITY  + presence of a convenience store on home-to-school route  0 presence of a bakery/café/restaurant on home-to-school route  0 presence of small retail on home-to-school route  <> AESTHETICS  0 presence of vacant lot on home-to-school route  <> ACCESSIBILITY  0 presence of highway or freeway on home-to-school route  <> TRAFFIC SAFETY  **- presence of road with busy traffic on home-to-school route**  0 presence of intersection without street signals or stop signs on home-to-school route  0 presence of intersection without a painted crosswalk on home-to-school route  WALK/CYCLE FACILITIES  **0 sidewalk maintenance condition on home-to-school route**  0 sidewalk on home-to-school route wide enough  0 sidewalk on home-to-school route free of obstructions  TRAFFIC SAFETY  0 sidewalk on home-to-school route separated from traffic  ACCESSIBILITY  **+ convenience of walking to school**  AESTHETICS  0 well maintained and clean  0 well shaded by trees  + quiet neighborhood  + nice things to see  SAFETY  0 well-lit street  <> SAFETY  - Concerns about getting lost  0 concerns about being harmed or exhaust fumes  <> CRIME SAFETY  - concerns about being taken or hurt by a stranger  0 concerns about getting bullied, teased, or harassed  0 concerns about being attacked by stray dogs  0 concerns about no one to see and help my child in case of danger  <> TRAFFIC SAFETY  - concerns about being hit by a car  **Multivariate model** | * Data collected in 22 schools  * RR parents (22.7% - 34.2%) |
| (Leslie et al. 2010) | N=2961  10-14 yr (M=11.4±0.8 yr)  Australia | CS | Web-based questionnaire for students  area level SES was obtained by Australian Bureau of Statistics (ABS) | *Web-based questionnaire for children*  ATS to school | *Binary logistic regression models*  RECREATION FACILITIES  + recreational facilities close to home  SAFETY  + safe to walk/jog in neighborhood^b^  AESTHETICS  + high community disorder^b^ | *only children who lived within 2.0km from school were included in the analyses  * RR schools = 51 %  * RR students year 6: 92%  * RR students year 8: 89%  * controlled for: age, location, area SES, self described health status, enjoyment of PA, family support for PA, friend support for PA, sports teams available, scouting groups available, youth groups available, adequate equipment at home for PA  * community disorder= empty or abandoned buildings, graffiti, fights, crime and or drug selling, neighborhood safety |
| (Lin and Chang 2010) | N=330  1^st^-6^th^ grade  Taiwan | CS | - existing databases - GIS - audit | *Student survey*  WTS to school (walking dependently or independently) | *Two-level nested logit models*  LAND USE MIX DIVERSITY  + mixed land use  WALK/CYCLE FACILITIES  + sidewalk percentage  CONNECTIVITY   - intersection number   RESIDENTIAL DENSITY  0 building density | * Data collected in 3 schools  RR=91,68%  *children in this study did not use a bicycle  *mode option diversity = sum of normalized values of bus route number between home and school, having (=1) or having no (=0) vanpool service, car ownership, motorcycle ownership and bicycle ownership for a child household  *block size = average block size within the residence of area of a child  * existing databases and published documents: population data – land use data – data on employment, vehicle ownership & travel speed of various mode  * GIS: road area – road length – block size – shade trees – intersections – slope gradient  * audit: sidewalks – school characteristics |
| (Lin and Yu 2011) | n=382  10-12yr  Taiwan | CS | -existing databases | *children’s questionnaire*  walking to organized leisure activities on a weekday | *Multi-nomial logit model*  CONNECTIVITY  0 intersection density  <>TRAFFIC SAFETY  0 traffic density  WALK/CYCLE FACILITIES  0 walkway quality  0 walkway width  LAND USE MIX DIVERSITY  - mixed land use  RESIDENTIAL DENSITY  + building density  RECREATION FACILITIES  0 leisure facility supply | * RR=98.1% |
|  |  |  |  | *children’s questionnaire*  cycling to organized leisure activities on a weekday | *Multi-nomial logit model*  CONNECTIVITY  0 intersection density  <> TRAFFIC SAFETY  0 traffic density  WALK/CYCLE FACILITIES  0 walkway quality  0 walkway width  LAND USE MIX DIVERSITY  0 mixed land use  RESIDENTIAL DENSITY  0 building density  RECREATION FACILITIES  0 leisure facility supply |  |
|  |  |  |  | *children’s questionnaire*  walking to organized leisure activities on a weekendday | CONNECTIVITY  + intersection density  <> TRAFFIC SAFETY  - traffic density  WALK/CYCLE FACILITIES  + walkway quality  0 walkway width  LAND USE MIX DIVERSITY  0 mixed land use  RESIDENTIAL DENSITY  0 building density  RECREATION FACILITIES  0 leisure facility supply |  |
|  |  |  |  | *children’s questionnaire*  cycling to organized leisure activities on a weekendday | CONNECTIVITY  + intersection density  <> TRAFFIC SAFETY  - traffic density  <> WALK/CYCLE FACILITIES  + walkway quality  0 walkway width  LAND USE MIX DIVERSITY  + mixed land use  RESIDENTIAL DENSITY  0 building density  RECREATION FACILITIES  0 leisure facility supply |  |
|  |  |  |  | *children’s questionnaire*  walking to unorganized leisure activities on a weekday | CONNECTIVITY  + intersection density  <> TRAFFIC SAFETY  - traffic density  WALK/CYCLE FACILITIES  + walkway quality  - walkway width  LAND USE MIX DIVERSITY  0 mixed land use  RESIDENTIAL DENSITY  0 building density  RECREATION FACILITIES  0 leisure facility supply |  |
|  |  |  |  | *children’s questionnaire*  cycling to unorganized leisure activities on a weekday | CONNECTIVITY  0 intersection density  <> TRAFFIC SAFETY  0 traffic density  WALK/CYCLE FACILITIES  0 walkway quality  0 walkway width  LAND USE MIX DIVERSITY  0 mixed land use  RESIDENTIAL DENSITY  0 building density  RECREATION FACILITIES  0 leisure facility supply |  |
|  |  |  |  | *children’s questionnaire*  walking to unorganized leisure activities on a weekendday | CONNECTIVITY  + intersection density  <> TRAFFIC SAFETY  - traffic density  WALK/CYCLE FACILITIES  0 walkway quality  0 walkway width  LAND USE MIX DIVERSITY  - mixed land use  RESIDENTIAL DENSITY  + building density  RECREATION FACILITIES  0 leisure facility supply |  |
|  |  |  |  | *children’s questionnaire*  cycling to unorganized leisure activities on a weekendday | CONNECTIVITY  + intersection density  <> TRAFFIC SAFETY  - traffic density  WALK/CYCLE FACILITIES  0 walkway quality  0 walkway width  LAND USE MIX DIVERSITY  0 mixed land use  RESIDENTIAL DENSITY  0 building density  RECREATION FACILITIES  0 leisure facility supply |  |
| (Loucaides et al. 2010) | N=448  Grade 6, 11.8 ±0.4 years  Cyprus | CS | children’s questionnaire | *children’s questionnaire*  ATS | *bivariate logistic regression analyses*  CONNECTIVITY  - intersections  SAFETY  + feeling safe to walk  **+ my parents think it is safe** | * children were classified as active travelers to school if they reported usual mode of travel to school walk or bicycle and non)active travelers if they reported as usual mode of travel bus or car, and motorcycle |
| (Martin et al. 2007) | N=2579  9-15 yr (M=11.6yr)  USA | CS | Questionnaire for children’s perceptions | *Parental questionnaire*  ATS (=walking or biking to school one or more times per week in a usual week) | *Unadjusted and adjusted logistic regression*  RECREATION FACILITIES  + perception of a lot of places in the neighborhood to be physically active | * YMCLS = Youth Media Campaign Longitudinal Survey = a random-digit-dialed survey developed to evaluate the VERB campaign.  * RR= ±85%  * only children included who lived less than 1 mile from school  *adjusted for: age gender, race/ethnicity, region, parental level of education, household income, number of children in the household, marital status |
| (McDonald 2007) | N=614  5-18 yr (M=10.9±4yr)  USA: California | CS | GIS | WTS  (2 day activity diary completed by parents) | *Binary logit model*  RESIDENTIAL DENSITY  0 Dwelling units per sq km  LAND USE MIX DIVERSITY  + Land use mix | * children under 15 were proxy-reported by the parents  *only youth with trips to school of 1.6 miles or less were included in the models  *2000 Metropolitan Transportation Commission Bay Area Travel Survey (BATS) by parents for children <15yr |
| (McDonald 2008a) | N=14 553  5-18 yr  (M=11.4yr)  USA | CS | census | *2001 National Household Travel Survey*  ATS | *Binary model*  RESIDENTIAL DENSITY  + density: more ATS when density: 10 000- 24 999 people per sq. mile | * controlled for: distance to school, age, possession of drivers license, sex, annual household income, household vehicles per driver, built year house, density, neighborhood disadvantage, race  * RR= 34.1% |
| (McDonald 2008b) | n=6508  5-13 yr ( M: 9.3±2.5)  USA | CS | census | *2001 National Household Travel Survey*  WTS | *multinomial logit model results*  RESIDENTIAL DENSITY  + population density |  |
| (McMillan 2007) | 16 elementary schools  N=???  Grade 3-5  USA: California | CS | Caregiver’s survey of the perceived neighborhood +  audit data: observations on each street segment within a quarter mile radius of the elementary school | *Caregiver’s survey*  ATS (walk or bike vs. private vehicle or neighborhood carpool) | *Binomial logit regression probability models*  Parental survey (home neighborhood)  <> SAFETY  - neighborhood is not safe to walk  <> TRAFFIC SAFETY  - child would have to travel on a road with traffic >30 mph  Audit (school neighborhood):  CRIME SAFETY  + amounts of windows facing the streets  LAND USE MIX DIVERSITY  + mixed use  WALK/CYCLE FACILITIES  0 sidewalk on both sides of the street | * caregiver survey: mode of travel to school, neighborhood safety, traffic safety, household transportation options, social/cultural norms, attitudes, socio-demographics.  *audit data: proportion of street segments with a complete sidewalk, proportion of street segments with greater than 50% of houses containing windows facing the street, proportion of street segments with a mix of land uses within ¼ mile radius from school |
| (Merom et al. 2006) | N=812  5-12 yr  Australia: New South Wales | CS | Computer Assisted Telephone Interview (CATI) for parents | *Computer Assisted Telephone Interview (CATI) for parents*  Frequent ATS (5 or more trips) and regular ATS (10 trips (=all possible trips to en from school)) | *Bivariate logistic regression analyses*  <>TRAFFIC SAFETY  **- parental perception of unsafe roads to school**  **Multivariate logistic regression analysis** | * children and parents who took part in this study mostly lived in urban areas  * reported trips that included any walking or cycling were considered as active commuting  * RR=67% |
| (Mitra et al. 2010) | n=3767  11-13 yr (M=11.97)  Canada, Toronto | CS | Census/GIS | *proxy-reported CATI*  WTS (Home-to-school trips on a randomly selected weekday) | *Bivariate correlations*  Neighborhood environment  RESIDENTIAL DENSITY  + population density of the traffic analysis zone of a child’s residence  CONNECTIVITY  - number of 4-way local street intersections within a 400m radius of a child’s residence  + number of street blocks within a 400m radius of a child’s residence  **+ number of street blocks within a 400m radius of child’s residence**  School environment  RESIDENTIAL DENSITY  + population density of the traffic analysis zone of a child’s school  CONNECTIVITY  0 number of 4-way local street intersections within a 400m radius of a child’s residence  + number of street blocks within a 400m radius of a child’s school location  + number of street blocks within a 400m radius of child’s school location  **Binomial logit modeling** | * 2001 Transportation Tomorrow Survey (TTS) |
| (Mitra and Buliung 2011) | n=2190  11-12yr (M:11.53yr)  Canada, Toronto | CS | Census/GIS | *2006 Transportation Tomorrow Survey filled out by an adult housemember*  ATS 800 m home buffer | *Multivariate logistic regression*  CONNECTIVITY  0 fourway density  0 block density  SAFETY  0 light density | * only children living within 5km (shortest path network distance) from their schools were considered for modeling purposes (=92% of all school trips by this age group) |
| (Mitra and Buliung 2014) | N=945  11-year-old children  Canada, Toronto | CS | Census/GIS home neighborhood | *2006 Transportation Tomorrow Survey filled out by an adult housemember*  WTS | *Multinomial logit models*  TRAFFIC SAFETY  + child did not have to cross a major street on the way to school  CONNECTIVITY  - street connectivity  + number of street-blocks within a 400 buffer  <> STREET CONNECTIVITY  0 proportion of dead end and cul-de-sacs  LAND USE MIX DIVERSITY  + mix retail  TRAFFIC SAFETY  0 proportion of intersections that are signalized | * 2006 Transportation Tomorrow Survey  * only children living within 3.2 km from school |
|  |  |  | Census/GIS school neighborhood | *2006 Transportation Tomorrow Survey filled out by an adult housemember*  WTS | *Multinomial logit models*  TRAFFIC SAFETY  + child did not have to cross a major street on the way to school  CONNECTIVITY  - street connectivity  0 number of street-blocks within a 400 buffer  <> STREET CONNECTIVITY  0 proportion of dead end and cul-de-sacs  LAND USE MIX DIVERSITY  + mix retail  TRAFFIC SAFETY  0 proportion of intersections that are signalized |  |
| (Noland et al. 2012) | N=1571,  Mean grade = 3.53,  New Jersey, USA | CS | GIS | *Parental survey*  WTS | *Mixed logit model (random coefficients)*  RESIDENTIAL DENSITY  + residential density around school  CONNECTIVITY  + grid network around school  - block size around school  WALK/CYCLE FACILITIES  + connectivity of sidewalks around school  TRAFFIC SAFETY  0 speed limits of 25 mph | * Data collected in 19 schools |
| (Napier et al. 2011) | n=193 children, 177 parents  5^th^ grade  USA | CS | census | *children’s report*  WTS | *Multivariate generalized estimating equation*  WALKABILITY  + walkability | * RR parents=62.99% |
| (Oluyomi et al. 2014) | N=830 (81 elementary schools),  4^th^ grade students, Texas, USA | CS | Parental and children’s survey | *Parental survey*  WTS (usual transport mode) | *Bivariate logistic regression analysis*  Traffic safety home environment  WALK/CYCLE FACILITIES  **+Sidewalks on most of neighborhood streets**  AESTHETICS  **+Sidewalks in neighborhood well-maintained**  TRAFFIC SAFETY  **+Safe road crossings in your neighborhood**  Traffic safety environment en-route to school  <> TRAFFIC SAFETY  **-Amount of traffic along route a problem**  **-Safety at intersections and crossings a problem**  **-Crossing guards a problem**  <> WALK/CYCLE FACILITIES  **-Sidewalks or pathways a problem**  Traffic safety school environment  WALK/CYCLE FACILITIES  **+Sidewalks on streets near child’s school**  AESTHETICS  0 sidewalks well-maintained  0 trees along streets near school  0 bike lanes/paths or trails well maintained  TRAFFIC SAFETY  **+ safe road crossings**  Personal safety home environment  SAFETY  0 feeling safe walking in the neighborhood (children’s perception)  0 feeling safe riding a bike in the neighborhood (children’s perception)  **+ safe for child to walk/bike in neighborhood**  <> CRIME SAFETY  0 afraid when out alone after dark  Personal safety (en-route)  CRIME SAFETY  **+ adults or other children to bike/with**  <> CRIME SAFETY   - **violence or crime a problem** - **stray or dangerous animals a problem**   Personal safety (school)  AESTHETICS  0 attractive things to look at  0 well-maintained homes, apartments and gardens  <> AESTHETICS  0 abandoned houses or vacant lots  0 condoms, drug-related paraphernalia (needles, syringes, etc.)  **Multivariable model adjusted for student’s ethnicity, any type of public assistance (family), car ownership (family)** | * Texas Childhood Obesity Prevention Policy Evaluation (T-COPPE) project  * only children living within 2 mile from school |
| (Pabayo et al. 2012) | N= 710  6-8 yr  Canada, Québec | L | Parental report (structured interview) | *parental questionnaire*  ATS (usual travel mode) | *Multilevel growth curve models*  <> AESTHETICS  + decay | * Québec Longitudinal Study of Child Development (QLSCD)  * parents were asked if each of the following problems in their neighborhood was a problem (grave problem to not a problem): garbage, trash or broken glass; the sale or consumption of drugs; the presence of alcohol or excessive consumption of alcohol in public; groups of youths causing problems |
| (Page et al. 2010) | N=1307  10-11 yr  UK | CS | Computorised questionnaire for children | *Computorised questionnaire* *for children*  ATS (how do you usually get home from school: walking or going by bike vs. travel by car) | *Logistic regression modeling*  *BOYS:*  AESTHETICS  0 Aesthetics  SAFETY  0 Personal safety  TRAFFIC SAFETY  0 Traffic safety  LAND USE MIX DIVERSITY  + Access (1)  ACCESSIBILITY  + Acces (2)  RECREATION FACILITIES  0 Playspace  GIRLS:  AESTHETICS  0 Aesthetics  SAFETY  0 Personal safety  TRAFFIC SAFETY  0 Traffic safety  LAND USE MIX DIVERSITY  0 Access (1)  ACCESSIBILITY  0 Acces (2)  RECREATION FACILITIES  0 Playspace | *the primary schools in this study were located in relatively deprived areas  * RR schools = 23/24 = 95.8%  * RR=70.5%  * baseline date from the ‘Personal and Environmental Associations with Children’s Health’ (PEACH) project  * adjusted for: minutes of daylight after school, BMI, level of neighborhood deprivation, pubertal status  * acces (1) = access to local shops, big shopping centre, playground or open space, bus stop, sports centre and library  Acces (2): ease of access to school and best friend’s house  * local IM = best friend’s house, school, local shops, and park or playground  Area-IM = swimming pool, library, cinema, arcade, bus stop, sports and shopping centre  *nuisance=perceptions of crime, noise, bullying in local neighborhood  *personal safety = perceptions of safety at night, daytime, fear of strangers, dark  * traffic safety= perceptions of safe places to cross, heavy traffic, roads, pollution  * Constraint = the child’s perception that they were restricted from being active by their physical environment |
| (Panter et al. 2010a) | N= 2012  9-10 yr  UK: Norfolk | CS | GIS (home environment + route to school)  School audit (school environment) | *Questionnaire for children*  WTS (walking) | *Multinomial regression analysis*  Neighborhood characteristics  CONNECTIVITY  + road density  RESIDENTIAL DENSITY  0 Building density  SAFETY  0 Streetlight density  <>TRAFFIC SAFETY  0 Traffic accidents per kilometer of roads  WALK/CYCLE FACILITIES  0 Pavement density  + Effective walkable area  CONNECTIVITY  - Connected node ratio  - Junction density  LAND USE MIX DIVERSITY  0 Land use mix  Route characteristics  SAFETY  0 Streetlight density  <>TRAFFIC SAFETY  0 Traffic accidents per kilometer en route  LAND USE MIX DIVERISTY  0 Land use mix  School characteristics  LAND USE MIX DIVERSITY  0 Land use mix around school (single=ref)  WALK/CYCLE FACILITIES  - Pavements  TRAFFIC SAFETY  0 Traffic calming  WALK/CYCLE FACILITIES  0 Pedestrian crossing  **Multivariate model** | * RR schools: 58.8% (92 schools included)  RR students: 57.0%  * Sport, Physical activity and Eating Behaviours: Environmental Determinants in Young people (SPEEDY study)  * Travel plan = a formal document, which identifies ways to encourage walking, cycling, or use of public transport)  * effective walkable area= total neighborhood area (the area that can be reached via the street network within 800 m from the home) divided by the potential walkable area (the area generated using a circular buffer with a radius of 800m from the home)  * connected node ratio = connectivity = number of junctions divided by number of junctions and cul-de-sacs  *route length ratio = route length divided by the straight line distance between the home and school  * analyses adjusted for: child age, gender, BMI, parental car access, maternal travel mode to work, and journey length |
|  |  |  |  | *Questionnaire for children*  CTS cycling | *Multinomial regression analysis*  Neighborhood characteristics  CONNECTIVITY  0 road density  RESIDENTIAL DENSITY  0 Building density  SAFETY  - Streetlight density  <> TRAFFIC SAFETY  0 Traffic accidents per kilometer of roads  WALK/CYCLE FACILITIES  0 Pavement density  0 Effective walkable area  CONNECTIVITY  0 Connected node ratio  0 Junction density  LAND USE MIX DIVERSITY  - Land use mix  Route characteristics  SAFETY  - Streetlight density  <> TRAFFIC SAFETY  0 Traffic accidents per kilometer en route  LAND USE MIX DIVERSITY  - Land use mix  School characteristics  LAND USE MIX DIVERISTY  0 Land use mix around school (single=ref)  WALK/CYCLE FACILITIES  0 Pavements  TRAFFIC SAFETY  0 Traffic calming  WALK/CYCLE FACILITIES  0 Pedestrian crossing  **Multivariate model** |  |
| (Panter et al. 2010b) | N=2012 (n=760 for children living <1 km from school)  9-10 yr  UK: Norfolk | CS | GIS (distance to school and urban rural status)  parental and children’s surveys | *Questionnaire for children*  WTS (usual travel mode) | *Multilevel logistic regression*  Distance to school < 1 km:  *Route environment (parental perception)*  <> TRAFFIC SAFETY  - Traffic concern  <> SAFETY  - Concern about something happening to my child  *Neighborhood environment (children’s perception)*  SAFETY  + Safe to walk or play in my neighborhood during the day  <> SAFETY  0 Difficult to walk or play near my house, because I don’t feel safe  *Neighborhood environment (parental perception)*  WALKABILITY  + Neighborhood walkability score | * RR schools: 58.8%  RR students: 57.0%  * Sport, Physical activity and Eating Behaviours: Environmental Determinants in Young people (SPEEDY study)  * analyses adjusted for: child age, gender, BMI, parental car access, maternal travel mode to work, and journey length |
|  |  |  |  | *Questionnaire for children*  CTS (usual travel mode) | Distance to school < 1 km:  *Route environment (parental perception)*  <> TRAFFIC SAFETY  - Traffic concern  <> SAFETY  - Concern about something happening to my child  *Neighborhood environment (children’s perception)*  SAFETY  0 Safe to walk or play in my neighborhood during the day  <> SAFETY  0 Difficult to walk or play near my house, because I don’t feel safe  *Neighborhood environment (parental perception)*  WALKABILITY  + Neighborhood walkability score |  |
| (Panter et al. 2013) | N=912  Mean age: 10.2±0.3 years (+1 year follow up), Norfolk UK | L | Objective methods  Parental en children’s perceptions | *Children’s questionnaire*  Uptake of ATS (vs. maintaining passive transportation) | *Logistic regression analysis*  Children’s perceptions of the neighborhood:  TRAFFIC SAFETY  **+ Composite of route safety**  SAFETY  0 Safe to walk/play in alone in my neighbourhood during the day  Objective environment:  CONNECTIVITY  + Road density   - Junction density   SAFETY  + Street light density  + Streetlight density on route  WALK/CYCLE FACILITIES   - Effective walkable area   LAND USE MIX DIVERSITY   - Land use mix   **Multivariate model** | * Sport and Physical activity and Eating behaviour: environmental determinants in young people (SPEEDY)  * RR follow up: 44.2% |
|  |  |  |  | *Children’s questionnaire*  maintaining ATS (vs. taking up passive transportation) | *Logistic regression analysis*  Children’s perceptions of the neighborhood:  TRAFFIC SAFETY  + Composite of route safety  SAFETY  0 Safe to walk/play in alone in my neighbourhood during the day  Objective environment:  CONNECTIVITY  0 Road density  0 Junction density  SAFETY  0 Street light density  0 Streetlight density on route  WALK/CYCLE FACILITIES  0 Effective walkable area  LAND USE MIX DIVERSITY  0 Land use mix  **Multivariate model** |  |
| (Rodriguez and Vogt 2009) | N=1897  7-13 yr  USA: Michigan | CS | Michigan Safe Routes to School Student Survey | *Michigan Safe Routes to School Student Survey*  WTS | *spearman correlation*  TRAFFIC SAFETY   - Safe route infrastructure   SAFETY  **+ perception of walking is safe**  **Logistic regression** | * 11 elementary schools  * RR = 84% |
| (Rosenberg et al. 2009) | n=116  5-11 yr (M=8.3 yr)  USA: Boston, Cincinatti, San Diego | CS | NEWS-Y completed by parents | *parental report*  walking to a park once per week or more | *One-way analysis of covariance*  LAND USE MIX DIVERSITY  0 land use mix-diversity  TRAFFIC SAFETY  0 pedestrian and automobile traffic safety  CRIME SAFETY  0 crime safety  AESTHETICS  0 aesthetics  WALK/CYCLE FACILITIES  0 walking and cycling facilities  CONNECTIVITY  0 street connectivity  LAND USE MIX ACCESSIBILITY  + land use mix-access  RESIDENTIAL DENSITY  0 residential density  RECREATION FACILITIES  + recreation facilities | * San Diego: 54% RR ‘cold calling method’ San Diego: 15% RR community centers  Cincinnati: 73% contacted by study introductory letter  Boston: 47.5%: face-to-face contact and phone prompting |
|  |  |  |  | *parental report*  walking to shops once per week or more | *One-way analysis of covariance*  LAND USE MIX DIVERSITY  + land use mix-diversity  TRAFFIC SAFETY  0 pedestrian and automobile traffic safety  CRIME SAFETY  + crime safety  AESTHETICS  0 aesthetics  WALK/CYCLE FACILITIES  + walking and cycling facilities  CONNECTIVITY  + street connectivity  LAND USE MIX ACCESSIBILITY  + land use mix-access  RESIDENTIAL DENSITY  0 residential density  RECREATION FACILITIES  + recreation facilities |  |
|  |  |  |  | *parental report*  walking to/from school once per week or more | *One-way analysis of covariance*  LAND USE MIX DIVERSITY  + land use mix-diversity  TRAFFIC SAFETY  0 pedestrian and automobile traffic safety  CRIME SAFETY  0 crime safety  AESTHETICS  0 aesthetics  WALK/CYCLE FACILITIES  0 walking and cycling facilities  CONNECTIVITY  0 street connectivity  LAND USE MIX ACCESSIBILITY  0 land use mix-access  RESIDENTIAL DENSITY  + residential density  RECREATION FACILITIES  0 recreation facilities |  |
| (Rossen et al. 2011) | n=365  8-13 yr (M=9.60±1.04yr)  USA, Baltimore | CS | Audit (incivilities)  Children’s children’s perception (safety) | *Children’s report*  WTS | *Univariate logistic regression models*  SAFETY  0 neighborhood safety  <>AESTHETICS  **+ incivilities**  **Multivariate logistic regression model** | * Multiple Opportunities to Reach Excellence (MORE) project  * incivilities: physical disorder, social disorder, violence, alcohol, and drug indicators |
| (Rothman et al. 2014) | 118 elementary schools,  Toronto, Canada | CS | GIS (density and diversity measures around school)  Audits (design measures around school) | *Observations*  WTS | *Negative binomial regression*  DENSITY  + total population (number)/1000m²  RECREATION FACILITIES  + recreational facilities  0 park land area/boundary  LAND USE MIX DIVERSITY  0 mixed land use  TRAFFIC SAFETY  **+ school crossing guard observed**  + crossing guards/km roads  0 traffic calming segment km/km roads  **+ traffic light/km roads**  0 flashing lights/km roads  <> TRAFFIC SAFETY  0 cars appear to be driving fast near school  0 dangerous midblock crossing near school  0 dangerous intersection near school  0 mean speed>5km over speed limit  0 school traffic/minute  <> AESTHETICS  0 old houses  <> CONNECTIVITY  0 dead end (number/km roads)  CONNECTIVITY  **+ intersection (number/km roads)**  0 route connectivity (intersections/dead ends)  WALK/CYCLE FACILITIES  **+ pedestrian crossover/km roads**  0 sidewalks missing km/km road  **Multivariate model** | * RR schools = 118/126 = 93.7% |
| (Salmon et al. 2007) | n=366  4-13 yr  Australia | CS | CATI parental survey | *CATI parental survey*  ATS (≤15-minute walk from school)  (n=366) | *Separate logistic regression model*  <> TRAFFIC SAFETY  0 I’m concerned about my child might be injured in a road accident walking to school  0 most drivers exceed the speed limits in my nearby streets  - too much traffic in our neighborhood  <> WALK/CYCLE FACILITIES  0 not enough pedestrian crossings or lights for my child to use  0 there are no footpaths in my neighborhood  <> ACCESSIBILITY  **- no direct route for my child to walk to school**  <> SAFETY  - our neighborhood is not safe for my child to walk to school | * RR= 27%  * study based on data collected as part of the evaluation of the Pedestrian Council of Australia’s National Walk Safely to School Day  * analyses are adjusted for: age, sex and SES  * variables in bold remained significant in multiple models  * Only children included living ≤15 minutes walking from school |
| (Steinbach et al. 2012) | N= 4513 (for analyses during term time) and n=3569 (for analyses during the summer and weekends)  5-17 yr (M:11yr)  United Kingdom | CS | GIS neighborhood | *Interview: one day travel diary*  WTS | *Logistic regression*  <>TRAFFIC SAFETY  + traffic speed  - traffic volume  CONNECTIVITY  0 number of junctions | * London Travel Demand Survey |
|  |  |  |  | *Interview one day travel diary*  Some walking weekday (>100m, not WTS) | *Logistic regression*  <>TRAFFIC SAFETY  0 traffic speed  0 traffic volume  CONNECTIVITY  - number of junctions |  |
|  |  |  |  | *Interview one day travel diary*  Some walking (>100m, summer and weekend travel) | *Logistic regression*  <> TRAFFIC SAFETY  - traffic speed  + traffic volume  CONNECTIVITY  - number of junctions |  |
| (Su et al. 2013) | N=4338,  5-7 year old (mean age: 6.6 yrs), Los Angeles, USA | CS | GIS | *Parental questionnaire*  WTS | *Bivariate logistic analysis*  <> TRAFFIC SAFETY   - **Traffic density around home**   LAND USE MIX DIVERSITY  **+ Land use mix around home**  0 Simpson’s diversity index around home  CONNECTIVITY   - **Connectivity around home**   <> TRAFFIC SAFETY  0Traffic density around school  LAND USE MIX DIVERSITY  0 Land use mix around school  0 Simpson’s diversity index around school  CONNECTIVITY  0 Connectivity around school  **Multiple logistic analysis** | * Children Health Study (CHS)  * RR=65%  * 500 m buffer distance was used (300 m for traffic density) |
| (Timperio et al. 2004) | n=919  10-12 yr  Australia: Melbourne | CS | parental and children’s questionnaire | *parental questionnaire*  walking / cycling to destinations at least three times a week | *Unconditional logistic regression analysis*  *BOYS*  parental perceived characteristics  <>TRAFFIC SAFETY  - no lights/crossings  - Have to cross several roads  0 road safety is a concern  0 heavy traffic in our local streets  <> RECREATION FACILITIES  0 Few sporting venues  <> CRIME SAFETY  0 stranger danger is a concern  Children’s perceived characteristics  <>RECREATION FACILITIES  - No parks near where live  <> TRAFFIC SAFETY  0 I think there is heavy traffic in the streets where I live  0 My Mum and Dad think there is heavy traffic in our streets  <> CRIME SAFETY  0 I am worried about strangers  0 My Mum and Dad are worried about strangers  TRAFFIC SAFETY  0 I think the roads are safe  0 My Mum and Dad think the roads are safe  *GIRLS*  parental perceived characteristics  <>TRAFFIC SAFETY  0 no lights/crossings  - Have to cross several roads  0 road safety is a concern  0 heavy traffic in our local streets  <> RECREATION FACILITIES  - Few sporting venues  <> CRIME SAFETY  0 stranger danger is a concern  Children’s perceived characteristics  <>RECREATION FACILITIES  - No parks near where live  <> TRAFFIC SAFETY  0 I think there is heavy traffic in the streets where I live  0 My Mum and Dad think there is heavy traffic in our streets  <> CRIME SAFETY  0 I am worried about strangers  0 My Mum and Dad are worried about strangers  TRAFFIC SAFETY  0 I think the roads are safe  0 My Mum and Dad think the roads are safe | * RRschools=79.2%  RR=44%  * Analyses were conducted separately for boys and girls |
| (Timperio et al. 2006) | n=919  10-12 yr  Australia: Melbourne | CS | parental and children’s questionnaire + GIS | *parental questionnaire*  ATS | *Bivariate logistic regression analysis*  *Parent-perceived social/physical neighborhood*  <>TRAFFIC SAFETY  0 heavy local traffic  0 strong concern about road safety  - no lights/crossings  0 need to cross several roads  <> CRIME SAFETY  0 strong concern about strangers  *Child-perceived social/physical neighborhood*  <>TRAFFIC SAFETY  0 heavy local traffic  - parents perceive heavy local traffic  - roads not safe  - parents perceive roads as not safe  <> CRIME SAFETY  0 worried about strangers  0 parents worried about strangers  *Objective measures of route to school (GIS)*  <>TRAFFIC SAFETY  - busy road barrier along school route  - school route along busy road  ACCESSIBILITY  - direct route to school  <> ACCESSIBILITY  - steep road barrier en route to school | * RRschools=79.2%  RR=44% |
| (Trapp et al. 2011) | N=1197  Grade 5,6,7  Australia | CS | Parental and children’s questionnaire  GIS | *Children’s 5-day travel diary*  CTS | *Bivariate associations*  *BOYS*  Parental perceptions:  SAFETY  + neighborhood is safe enough to CTS  <> ACCESSIBILITY  0 hilly steeps  <> TRAFFIC SAFETY  - child has to cross busy road  - no safe crossings for my child to use  - lot of traffic near school  0 drivers near school often exceed speed limit  <> WALK/CYCLE FACILITIES  0 lack of footpaths  Child perceptions  <> TRAFFIC SAFETY  0 I have to cross a busy road  TRAFFIC SAFETY  + I feel safe crossing the road near my school  <> TRAFFIC SAFETY  0 heavy traffic around school  0 heavy traffic around neighborhood  GIS (school environment)  WALKABILITY  + school walkability  <> TRAFFIC SAFETY  - school road traffic volume  CONNECTIVITY  + school street connectivity  *GIRLS*  Parental perceptions:  SAFETY  + neighborhood is safe enough to CTS  <> ACCESSIBILITY  - hilly steeps  <> TRAFFIC SAFETY  - child has to cross busy road  - no safe crossings for my child to use  - lot of traffic near school  - drivers near school often exceed speed limit  <> WALK/CYCLE FACILITIES  0 lack of footpaths  Child perceptions  <> TRAFFIC SAFETY  + I have to cross a busy road  0 I feel safe crossing the road near my school  0 heavy traffic around school  0 heavy traffic around neighborhood  GIS (school environment)  WALKABILITY  0 school walkability  <> TRAFFIC SAFETY  0 school road traffic volume  CONNECTIVITY  0 school street connectivity | * TRavel, Environment and Kids (TREK) project  RR children = 57%  RR parents = 88.8% |
| (Trapp et al. 2012) | N=617 boys and 681 girls,  Mean age: 11.0±0.8 years, Perth, Australia | CS | Parental and children’s survey  GIS | *Children’s 1 week travel diary*  WTS( < 6trips / week vs. ≥6 trips / week) | *Bivariate associations BOYS*  Parental perceptions  SAFETY  + Neighborhood is safe enough for children to walk to school with friends  <> WALK/CYCLE FACILITIES  0 There are not enough footpaths  <> TRAFFIC SAFETY   - My child would have to cross a busy road - There are no safe crossings for my child to use - There is a lot of traffic in the neighborhood   0 speeding traffic near school  <> CRIME SAFETY  0 fear of stranger danger  Child perceptions:  <> TRAFFIC SAFETY   - **I would have to cross a busy road**   - There is a lot of traffic in my neighborhood  0 perceived heavy traffic around school  0 perceived heavy traffic in the neighborhood  TRAFFIC SAFETY  0 I feel safe crossing the road near my school  <> CRIME SAFETY  0 fear of stranger danger  GIS (school environment):  WALKABILITY  0 school neighborhood walkability  <>TRAFFIC SAFETY  0 school traffic volume  CONNECTIVITY  0 school connectivity  **Multivariate logistic regression model** | * Travel, Environment and Kids (TREK) project  * Data collected in 25 schools |
|  |  |  |  |  | *Bivariate associations GIRLS*  Parental perceptions  SAFETY  + Neighborhood is safe enough for children to walk to school with friends  <> WALK/CYCLE FACILITIES   - There are not enough footpaths   <> TRAFFIC SAFETY   - My child would have to cross a busy road - There are no safe crossings for my child to use - There is a lot of traffic in the neighborhood   0 speeding traffic near school  <> CRIME SAFETY  0 fear of stranger danger  Child perceptions:  <> TRAFFIC SAFETY   - I would have to cross a busy road   0 There is a lot of traffic in my neighborhood  0 perceived heavy traffic around school  0 perceived heavy traffic in the neighborhood  TRAFFIC SAFETY  + I feel safe crossing the road near my school  CRIME SAFETY  0 fear of stranger danger  GIS:  WALKABILITY  + school neighborhood walkability  <>TRAFFIC SAFETY   - school traffic volume   CONNECTIVITY  0 school connectivity  **Multivariate logistic regression model** |  |
| (Zhu et al. 2008) | n=1281, 8  elementary school  USA: Texas | CS | parental survey | *parental survey*  WTS | *Binary logistic regressions*  <> SAFETY  **- safety concerns**  <> LAND USE MIX ACCESSIBILITY  **physical barrier:**  **- highway or freeway**  - busy road  WALK/CYCLE FACILITIES  + sidewalk quality  + quality of overall walking environment  LAND USE MIX DIVERSITY  **- convenience store en route to school**  **Multivariate analyses** | * focus on schools with high percentages of low-income families and Hispanic students  * RR = 26.9% |
| (Zhu and Lee 2009) | n=2695  19 elementary schools  USA: Texas | CS | parental survey | *parental survey*  WTS | *Bivariate logistic regression*  <> SAFETY  **- safety concerns**  <> LAND USE MIX ACCESSIBILITY  **Physical barrier:**  **-my child has to cross highway/freeway**  - my child has to cross busy road  <> TRAFFIC SAFETY  **- my child has to cross an intersection without a painted crosswalk**  WALK/CYCLE FACILITIES  + sidewalk quality  + quality of overall walking environment  LAND USE MIX DIVERSITY  **Land uses and public transport en route to school**  **- convenience store**  -bakery/café/restaurant  ***Multivariate logistic regression models*** | * RR=22.7%  * the use of walking as a typical mode of travel to/from school  * multivariate logistic regression model adjusted for: personal sociodemographic factors (gender, grade level, ethnicity, parental education level, single-parent status, number of family members and household’s car ownership), personal attitudes and behaviors (parents’ personal barriers, child’s personal barriers, parents’ and children’s positive attitudes and regular walking behavior), social factors (school bus availability, positive peer influence), school membership  * safety concerns:  - my child may get lost  - my child may be taken or hurt by a stranger  - my child may get bullied, teased or harassed  - my child may be attacked by stray dogs  - my child may be hit by a car  - exhaust fumes will harm my child’s health  * sidewalk quality:  - sidewalks are well maintained and clean  - sidewalks are wide enough  - sidewalks are separated from traffic by grass/trees  * walking environment  - it is convenient to walk to school  - it is well maintained and clean  - it is well shaded by trees  - it is quiet  - streets are well lit |

bold variables were also significant in a multivariable model

+ positive association was found between the physical environmental factor and active transportation

- negative association was found between the physical environmental factor and active transportation

0 no association was found between the physical environmental factor and active transportation

References

AARTS, M. J., MATHIJSSEN, J. J. P., VAN OERS, J. A. M. & SCHUIT, A. (2013) Associations Between Environmental Characteristics and Active Commuting to School Among Children: a Cross-sectional Study. *International Journal of Behavioral Medicine*, 20, (4) 538-555.

ALTON, D., ADAB, P., ROBERTS, L. & BARRETT, T. (2007) Relationship between walking levels and perceptions of the local neighbourhood environment. *Arch.Dis.Child*, 92, (1) 29-33.

BRAZA, M., SHOEMAKER, W. & SEELEY, A. (2004) Neighborhood design and rates of walking and biking to elementary school in 34 California communities. *AM J HEALTH PROMOT*, 19, (2) 128-136.

BRINGOLF-ISLER, B., GRIZE, L., M+ÑDER, U., RUCH, N., SENNHAUSER, F. H. & BRAUN-FAHRLANDER, C. (2008) Personal and environmental factors associated with active commuting to school in Switzerland. *Preventive Medicine*, 46, (1) 67-73.

CARSON, V., KUHLE, S., SPENCE, J. C. & VEUGELERS, P. J. (2010) Parents' perception of neighbourhood environment as a determinant of screen time, physical activity and active transport. *Canadian Journal of Public Health*, 101, (2) 124-127.

CARVER, A., TIMPERIO, A., HESKETH, K. & CRAWFORD, D. (2010) Are safety-related features of the road environment associated with smaller declines in physical activity among youth? *J Urban Health*, 87, (1) 29-43.

CARVER, A., TIMPERIO, A. F. & CRAWFORD, D. A. (2008) Neighborhood road environments and physical activity among youth: The CLAN study. *Journal of Urban Health-Bulletin of the New York Academy of Medicine*, 85, (4) 532-544.

CHILLON, P., HALES, D., VAUGHN, A., GIZLICE, Z., NI, A. & WARD, D. S. (2014) A cross-sectional study of demographic, environmental and parental barriers to active school travel among children in the United States. *Int J Behav Nutr Phys Act*, 11, 61.

CHRISTIANSEN, L. B., TOFTAGER, M., SCHIPPERIJN, J., ERSBOLL, A. K., GILES-CORTI, B. & TROELSEN, J. (2014) School site walkability and active school transport - association, mediation and moderation. *Journal of Transport Geography*, 34, 7-15.

CURRIERO, F. C., JAMES, N. T., SHIELDS, T. M., GOUVIS, R. C., FURR-HOLDEN, C. D., COOLEY-STRICKLAND, M. & POLLACK, K. M. (2013) Exploring walking path quality as a factor for urban elementary school children's active transport to school. *J Phys.Act.Health*, 10, (3) 323-334.

CUTUMISU, N., BÉLANGER-GRAVEL, A., LAFERÉ, M., LAGARDE, F., LEMAY, J. F. & GAUVIN, L. (2013) Influence of area deprivation and perceived neighbourhood safety on active transport to school among urban Quebec preadolescents. *Canadian journal of public health= Revue canadienne de sante publique*, 105, (5) e376-e382.

D'HAESE, S., DE MEESTER F., DE BOURDEAUDHUIJ, I., DEFORCHE, B. & CARDON, G. (2011) Criterion distances and environmental correlates of active commuting to school in children. *Int.J Behav.Nutr.Phys.Act.*, 8, 88.

D'HAESE, S., VAN DYCK, D., DE BOURDEAUDHUIJ, I., DEFORCHE, B. & CARDON, G. (2014) The association between objective walkability, neighborhood socio-economic status, and physical activity in Belgian children. *International Journal of Behavioral Nutrition and Physical Activity*, 11, 104.

DE VRIES, S. I., HOPMAN-ROCK, M., BAKKER, I., HIRASING, R. A. & VAN, M. W. (2010) Built environmental correlates of walking and cycling in Dutch urban children: results from the SPACE study. *Int.J Environ.Res Public Health*, 7, (5) 2309-2324.

DEWEESE, R. S., YEDIDIA, M. J., TULLOCH, D. L. & OHRI-VACHASPATI, P. (2013) Neighborhood perceptions and active school commuting in low-income cities. *Am.J Prev.Med.*, 45, (4) 393-400.

DUCHEYNE, F., DE BOURDEAUDHUIJ, I., SPITTAELS, H. & CARDON, G. (2012) Individual, social and physical environmental correlates of 'never' and 'always' cycling to school among 10 to 12 year old children living within a 3.0 km distance from school. *Int.J Behav.Nutr.Phys.Act.*, 9, 142.

DURAND, C. P., DUNTON, G. F., SPRUIJT-METZ, D. & PENTZ, M. A. (2012) Does community type moderate the relationship between parent perceptions of the neighborhood and physical activity in children? *AM J HEALTH PROMOT*, 26, (6) 371-380.

FRANK, L., KERR, J., CHAPMAN, J. & SALLIS, J. (2007) Urban form relationships with walk trip frequency and distance among youth. *American Journal of Health Promotion*, 21, (4) 305-311.

GALLIMORE, J. M., BROWN, B. B. & WERNER, C. M. (2011) Walking routes to school in new urban and suburban neighborhoods: An environmental walkability analysis of blocks and routes. *Journal of Environmental Psychology*, 31, (2) 184-191.

GILES-CORTI, B., WOOD, G., PIKORA, T., LEARNIHAN, V., BULSARA, M., VAN NIEL, K., TIMPERIO, A., MCCORMACK, G. & VILLANUEVA, K. (2011) School site and the potential to walk to school: The impact of street connectivity and traffic exposure in school neighborhoods. *Health & place*, 17, (2) 545-550.

HE, S. (2011) Effect of School Quality and Residential Environment on Mode Choice of School Trips. *Transportation Research Record* (2213) 96-104.

HSU, H. P. & SAPHORES, J. D. (2014) Impacts of parental gender and attitudes on children's school travel mode and parental chauffeuring behavior: results for California based on the 2009 National Household Travel Survey. *Transportation*, 41, (3) 543-565.

HUME, C., SALMON, J. & BALL, K. (2007) Associations of children's perceived neighborhood environments with walking and physical activity. *AM J HEALTH PROMOT*, 21, (3) 201-207.

HUME, C., TIMPERIO, A., SALMON, J., CARVER, A., GILES-CORTI, B. & CRAWFORD, D. (2009) Walking and Cycling to School Predictors of Increases Among Children and Adolescents. *American Journal of Preventive Medicine*, 36, (3) 195-200.

JOHANSSON, M. (2006) Environment and parental factors as determinants of mode for children's leisure travel. *Journal of Environmental Psychology*, 26, (2) 156-169.

KEMPERMAN, A. & TIMMERMANS, H. (2014) Environmental Correlates of Active Travel Behavior of Children. *Environment and Behavior*, 46, (5) 583-608.

KERR, J., ROSENBERG, D., SALLIS, J. F., SAELENS, B. E., FRANK, L. D. & CONWAY, T. L. (2006) Active commuting to school: associations with environment and parental concerns. *Medicine & Science in Sports & Exercise*, 38, (4) 787-794.

KYTTA, A. M., BROBERG, A. K. & KAHILA, M. H. (2012) Urban environment and children's active lifestyle: softGIS revealing children's behavioral patterns and meaningful places. *Am.J Health Promot.*, 26, (5) e137-e148.

LAROUCHE, R., CHAPUT, J. P., LEDUC, G., BOYER, C., BELANGER, P., LEBLANC, A. G., BORGHESE, M. M. & TREMBLAY, M. S. (2014) A cross-sectional examination of socio-demographic and school-level correlates of children's school travel mode in Ottawa, Canada. *Bmc Public Health*, 14.

LAROUCHE, R., FAULKNER, G. & TREMBLAY, M. S. (2013) Correlates of Active School Transport Immediately Before and After the Transition from Primary to Secondary School: A Pilot-Study. *Journal of Applied Research on Children: Informing Policy for Children at Risk*, 4, (2) 4.

LARSEN, K., BULIUNG, R. N. & FAULKNER, G. E. (2013) Safety and School Travel: How Does the Environment Along the Route Relate to Safety and Mode Choice? *Transportation Research Record* (2327) 9-18.

LEE, C., ZHU, X., YOON, J. & VARNI, J. W. (2013) Beyond distance: children's school travel mode choice. *Ann.Behav.Med.*, 45 Suppl 1, S55-S67.

LESLIE, E., KREMER, P., TOUMBOUROU, J. W. & WILLIAMS, J. W. (2010) Gender differences in personal, social and environmental influences on active travel to and from school for Australian adolescents. *Journal of Science and Medicine in Sport*, 13, (6) 597-601.

LIN, J. J. & CHANG, H. T. (2010) Built environment effects on children's school travel in Taipai: independence and travel mode. *Urban studies*, 47, (4) 867-889.

LIN, J. J. & YU, T. P. (2011) Built environment effects on leisure travel for children: Trip generation and travel mode. *Transport Policy*, 18, (1) 246-258.

LOUCAIDES, C. A., JAGO, R. & THEOPHANOUS, M. (2010) Prevalence and correlates of active traveling to school among adolescents in Cyprus. *Cent.Eur.J Public Health*, 18, (3) 151-156.

MARTIN, S. L., LEE, S. M. & LOWRY, R. (2007) National prevalence and correlates of walking and bicycling to school. *American Journal of Preventive Medicine*, 33, (2) 98-105.

MCDONALD, N. C. (2008a) Critical factors for active transportation to school among low-income and minority students. Evidence from the 2001 National Household Travel Survey. *Am.J Prev.Med.*, 34, (4) 341-344.

MCDONALD, N. C. (2007) Travel and the social environment: Evidence from Alameda County, California. *Transportation Research Part D: Transport and Environment*, 12, (1) 53-63.

MCDONALD, N. C. (2008b) Children's mode choice for the school trip: the role of distance and school location in walking to school. *Transportation*, 35, (1) 23-35.

MCMILLAN, T. E. (2007) The relative influence of urban form on a child's travel mode to school. *Transportation Research Part A: Policy and Practice*, 41, (1) 69-79.

MEROM, D., TUDOR-LOCKE, C., BAUMAN, A. & RISSEL, C. (2006) Active commuting to school among NSW primary school children: implications for public health. *HEALTH PLACE*, 12, (4) 678-687.

MITRA, R. & BULIUNG, R. Built Environment Correlates of Active School Transportation: Neighborhood and the Modifiable Areal Unit Problem, p. 19p.

MITRA, R. & BULIUNG, R. (2014) The influence of neighborhood environment and household travel interactions on school travel behavior: an exploration using geographically-weighted models. *Journal of Transport Geography*, 36, 69-78.

MITRA, R., BULIUNG, R. & ROORDA, M. (2010) Built Environment and School Travel Mode Choice in Toronto, Canada. *Transportation Research Record: Journal of the Transportation Research Board* (2156) 150-159.

NAPIER, M. A., BROWN, B. B., WERNER, C. M. & GALLIMORE, J. (2011) Walking to school: Community design and child and parent barriers. *Journal of Environmental Psychology*, 31, (1) 45-51.

NOLAND, R., PARK, H., VON HAGEN, L. A. & CHATMAN, D. A Mode Choice Analysis of School Trips in New Jersey, p. 33p.

OLUYOMI, A. O., LEE, C., NEHME, E., DOWDY, D., ORY, M. G. & HOELSCHER, D. M. (2014) Parental safety concerns and active school commute: correlates across multiple domains in the home-to-school journey. *International Journal of Behavioral Nutrition and Physical Activity*, 11, (1) 32.

PABAYO, R. A., GAUVIN, L., BARNETT, T. A., MORENCY, P., NIKIEMA, B. & SEGUIN, L. (2012) Understanding the determinants of active transportation to school among children: evidence of environmental injustice from the Quebec Longitudinal Study of Child Development. *HEALTH PLACE*, 18, (2) 163-171.

PAGE, A., COOPER, A., GRIEW, P. & JAGO, R. (2010) Independent Mobility, Perceptions of the Built Environment and Children's Participation in Play, Active Travel and Structured Exercise and Sport: The PEACH Project. *International Journal of Behavioral Nutrition and Physical Activity*, 7, (17) 10p.

PANTER, J. R., JONES, A. P., VAN SLUIJS, E. M. & GRIFFIN, S. J. (2010a) Neighborhood, route, and school environments and children's active commuting. *Am J Prev Med*, 38, (3) 268-278.

PANTER, J. R., JONES, A. P., VAN SLUIJS, E. M. F. & GRIFFIN, S. J. (2010b) Attitudes, social support and environmental perceptions as predictors of active commuting behaviour in school children. *Journal of Epidemiology & Community Health*, 64, (1) 41-48.

PANTER, J., CORDER, K., GRIFFIN, S. J., JONES, A. P. & VAN SLUIJS, E. M. (2013) Individual, socio-cultural and environmental predictors of uptake and maintenance of active commuting in children: longitudinal results from the SPEEDY study. *International Journal of Behavioral Nutrition and Physical Activity*, 10, (1) 83.

RODRIGUEZ, A. & VOGT, C. A. (2009) Demographic, environmental, access, and attitude factors that influence walking to school by elementary school-aged children. *J SCH HEALTH*, 79, (6) 255-261.

ROSENBERG, D., DING, D., SALLIS, J. F., KERR, J., NORMAN, G. J., DURANT, N., HARRIS, S. K. & SAELENS, B. E. (2009) Neighborhood Environment Walkability Scale for Youth (NEWS-Y): reliability and relationship with physical activity. *Preventive Medicine*, 49, (2-3) 213-218.

ROSSEN, L. M., POLLACK, K. M., CURRIERO, F. C., SHIELDS, T. M., SMART, M. J., FURR-HOLDEN, C. & COOLEY-STRICKLAND, M. (2011) Neighborhood Incivilities, Perceived Neighborhood Safety, and Walking to School Among Urban-Dwelling Children. *Journal of Physical Activity & Health*, 8, (2) 262-271.

ROTHMAN, L., TO, T., BULIUNG, R., MACARTHUR, C. & HOWARD, A. (2014) Influence of social and built environment features on children walking to school: An observational study. *Preventive Medicine*, 60, 10-15.

SALMON, J., SALMON, L., CRAWFORD, D. A., HUME, C. & TIMPERIO, A. (2007) Associations among individual, social, and environmental barriers and children's walking or cycling to school. *AM J HEALTH PROMOT*, 22, (2) 107-113.

STEINBACH, R., GREEN, J. & EDWARDS, P. (2012) Look who's walking: Social and environmental correlates of children's walking in London. *HEALTH PLACE*, 18, (4) 917-927.

SU, J. G., JERRETT, M., MCCONNELL, R., BERHANE, K., DUNTON, G., SHANKARDASS, K., REYNOLDS, K., CHANG, R. & WOLCH, J. (2013) Factors influencing whether children walk to school. *Health & place*, 22, 153-161.

TIMPERIO, A., BALL, K., SALMON, J., ROBERTS, R., GILES-CORTI, B., SIMMONS, D., BAUR, L. A. & CRAWFORD, D. (2006) Personal, family, social, and environmental correlates of active commuting to school. *American Journal of Preventive Medicine*, 30, (1) 45-51.

TIMPERIO, A., CRAWFORD, D., TELFORD, A. & SALMON, J. (2004) Perceptions about the local neighborhood and walking and cycling among children. *Preventive Medicine*, 38, (1) 39-47.

TRAPP, G. S., GILES-CORTI, B., CHRISTIAN, H. E., BULSARA, M., TIMPERIO, A. F., MCCORMACK, G. R. & VILLANEUVA, K. P. (2011) On your bike! a cross-sectional study of the individual, social and environmental correlates of cycling to school. *International Journal of Behavioral Nutrition and Physical Activity*, 8.

TRAPP, G. S. A., GILES-CORTI, B., CHRISTIAN, H., BULSARA, M., TIMPERIO, A., MCCORMACK, G. & VILLANEUVA, K. (2012) Increasing Children's Physical Activity: Individual, Social, and Environmental Factors Associated With Walking to and From School. *Health Education & Behavior*, 39, (2) 172-182.

ZHU, X., ARCH, B. & LEE, C. (2008) Personal, Social, and Environmental Correlates of Walking to School Behaviors: Case Study in Austin, Texas. *Thescientificworldjournal*, 8, 859-872.

ZHU, X. & LEE, C. (2009) Correlates of Walking to School and Implications for Public Policies: Survey Results from Parents of Elementary School Children in Austin, Texas. *Journal of Public Health Policy*, 30, (S1) S177-S202.
