# Supplementary material for: Cross-continental comparison of the association between the physical environment and active transportation in children: a systematic review
Source: Int J Behav Nutr Phys Act. 2015 Nov 26;12:145. doi: 10.1186/s12966-015-0308-z (PMC4660808; doi:10.1186/s12966-015-0308-z)
Supplement: Additional file 2: — Complete overview of the associations between the physical environment and physical activity. (DOCX 58 kb) [file 12966_2015_308_MOESM2_ESM.docx]

# Additional file 2. Complete overview of the associations between the physical environment and physical activity

# Relation between physical neighborhood environment and active transportation to school

|  | + | | | - | | | 0 | | | Active transportation to school | | |
| --- | --- | --- | --- | --- | --- | --- | --- | --- | --- | --- | --- | --- |
|  | Active transportation to school | Walking to school | Cycling to school | Active transportation to school | Walking to school | Cycling to school | Active transportation to school | Walking to school | Cycling to school | Active transportation to school | Walking to school | Cycling to school |
| WALKABILITY | **[42,42,67]** | [**56**,76,**80,87**] | [76,86] |  |  |  | [68,**69**] | **[87]^b^** | **[86]^g^** | 3/5  + | 4/5  + | 2/3  ? |
| density | **[42,49,73]** | [23,**50,54,54,55,60**] |  |  |  |  | [37,**40**,41,41,42,**66**] | **[48,77]** | [70,**77**] | 3/10  0 | 6/8  + | 0/2  ? |
| land use mix diversity | [42,*51*,75^b^] | [23,**45**,46,**48,52,52,61**] |  | [**29**,37] | [62,63,63] | **[77,77]** | [**29,42**,42,**43**,75^g^] | [46,46,**60,61,61,61,77,77**,*77*] | *[77]* | 3/10  0 | 7/20  (+) | 0/3  ? |
| street connectivity | [**29**,42] | [*15*,**52,54,54,54,54,55,60,77**] | **[86]^b^** | [**29**,74] | **[45,52,52,54,55,61,77,77]** |  | [*15,15*,**29,29**,30,30,37,37,37,**42,53,53**] | [*15*,23,**52,52,52,54,60,60,61,78,87^b^,87^g^**] | [*15,15*,70,**77,77,77,86^b^**] | 2/17  0 | 9/30  0 | 1/8  0 |
| ACCESSIBILITY | [42,68,75^b^,83,**85**] | [46,62,62,63,63] | [86]^g^ | **[85]** |  |  | [16,30,37,37,37,75] | [23,46] | [86]^b^ | 5/11  (+) | 6/8  + | 1/2  ? |
| WALK/CYCLE FACILITIES | [*15*,16,33,42,67] | [*15*,**34,34,45**,45,**55**,57,57,57,**60**,62,62,63,63,**77**,87^g^] | *[15]* | [**29**,*33*] | *[77]* |  | [15,15,29,30,30,36,37,43,43,43,43,43,51,68,83,83] | [*15,15*,23,46,46,46,46,**60**,64,**77**,*77*,87^b^] | [*15,15*,**64,70**,*77,77*,**77,77**,86^b^,86^b^] | 5/23  0 | 16/29  (+) | 1/11  0 |
| AESTHETICS | [36,42,81^b^] | [**45**,46,46,57] | *[15]* | [28,**73**] | [*15*,**34**,*59*,64,64] |  | [15,15,30,37,43,43,43,43,68,75,75,81] | [*15*,23,46,46,46,57,57,57,57,57,57,57,**60**,64] | [*15*,64,64,64,70] | 3/17  0 | 4/23  0 | 1/6  0 |
| SAFETY | [**29,29**,33,35,51,67,74,74,81^b^,83] | [34,45,46,57,58,62,63,76,76,87,87] | [76,86,86] |  |  | [*15*,**77,77**] | [16,29,29,29,29,35,53,66,75,75,81] | [34,34,45,46,46,57,57,59,76,**77,77**] | [*15,15*,76,76] | 10/21  (+) | 11/22  (+) | 3/10  0 |
| crime safety | *[51]* | [45,46,57,57,57] |  | [43] |  |  | [30,36,37,41,42,68,85,85,85] | [23,45,45,46,46,46,57,87,87,87,87] | [70] | 1/10  0 | 5/16  0 | 0/1  ? |
| traffic safety | [**29,29**,41,**43**,43,43,51,67,67,67,82,83,85,85,85,85,85,85] | [**45,45**,45,45,45,46,46,**52,52,**57,57,57,57,57,**60**,*60,60*,**61**,63,76,**78**,87^b^,87^b^,87^b^,87^b^,87^b^,87^g^,87^g^,87^g^,87^g^,87^g^,**87^g^**] | [64,70,76,**86**^b^,86^b^,86^b^,86^b^,86^b^,86^g^,86^g^,86^g^,86^g^] |  | [**45**,45,58,**78**] | [*15*,86^g^] | [*15,15,15*,30,30,30,36,37,41-43,43,**43,43**,**66**,68,75^b^,75^g^,83,83,85,85,85,85] | [*15,15,15*,23,45,45,45,45,46,46,46**,52,52,55,60,60**,*60,60,60,60,60*,**61,64**,*77*,**77,77**,87^g^,87^g^,87^g^,87^g^,87^b^,87^b^,87^b^,87^b^,**87^b^**] | [*15,15*,**77,77**,*77*,86^b^,86^b^,86^b^,86^b^,86^g^,86^g^,86^g^,**86^g^**] | 18/42  (+) | 32/71  (+) | 12/27  (+) |
| RECREATION FACILITIES | [16,47,81,81] | [46,**60**] | *[15]* |  |  |  | [*15,15*,75^b^,75^g^] | [*15,15*,23,46,**60**] | *[15]* | 4/8  (+) | 2/7  0 | 1/2  ? |

x/x = number of positive associations / number of total investigated associations

0 = 0-40% of the findings supporting the association = unrelated evidence

(+) or (-) = 41%–50% of the findings supporting the association = evidence for a possible association

+ or - = 51%–100% of the findings supporting a positive or negative association = convincing evidence

? = less than 3 variables were investigated

Bold: Using objective instruments to determine the physical environment

Italic: Using audit data to determine the physical environment

Underlined: using perceptions to determine the physical environment

b= in boys; g= in girls

# Relation between physical neighborhood environment and walking/cycling during leisure

|  | + | | | - | | | 0 | | | Active transportation during leisure | | |
| --- | --- | --- | --- | --- | --- | --- | --- | --- | --- | --- | --- | --- |
|  | Walking/cycling during leisure | Walking during leisure | Cycling during leisure | Walking/cycling during leisure | Walking/cycling during leisure | Walking during leisure | Cycling during leisure | Walking/cycling during leisure | Walking/cycling during leisure | Walking during leisure | Cycling during leisure | Walking/cycling during leisure |
| WALKABILITY |  |  |  |  | **[69]** |  |  | **[69]** | **[69]** | 0/0  ? | 0/2  ? | 0/1  ? |
| density |  | **[38,88,88]** |  |  |  |  |  | [23,23,**38,88,88**] | **[88,88,88,88]** | 0/0  ? | 3/8  0 | 0/4  ? |
| land use mix diversity |  | [23] | **[88]** |  | **[88,88]** |  |  | [23,**38,38,38,38,88,88**] | **[88,88,88]** | 0/0  ? | 1/10  0 | 1/4  ? |
| street connectivity |  | [*15*,23,**88,88,88**] | **[88,88]** |  | **[78,78]** |  | [*15,15*,**31,31,31,31,79,79**] | [*15*,23,**38,38,88**] | [*15,15*,**88,88**] | 0/8  0 | 5/12  (+) | 2/6  ? |
| ACCESSIBILITY |  | [23,23] |  |  |  |  |  |  |  | 0/0  ? | 2/2  ? | 0/0  ? |
| WALK/CYCLE FACILITIES | [*15*,**31**^g^] | [*15*,23**,88,88**] | [*15***,88**] |  | **[88]** | *[15]* | [*15,15*,**31**,71,**79**] | [*15,15*,23,**88,88,88,88,88**] | [*15*,**88,88,88,88,88,88,88**] | 2/7  0 | 4/13  0 | 2/11  ? |
| AESTHETICS |  |  |  |  |  |  | [*15,15*,**72**] | [*15,15*,23,23] | *[15,15]* | 0/3  ? | 0/4  ? | 0/2  ? |
| SAFETY |  |  |  |  |  |  | [71,72] |  |  | 0/2  ? | 0/0  ? | 0/0  ? |
| crime safety |  | [23,65] |  |  |  |  | [71,84,84,84,84,84,84] | [23,65] |  | 0/7  ? | 2/4  ? | 0/0  ? |
| traffic safety | [**31**,*71*,84^g^,84^b^,84^b^] | **[78,88,88,88]** | [*15*,**88,88**] |  | [*15*,65,65,65,65,**78**] | *[15]* | [*15,15,15*,**31,31,31,31,31,31,31**,71,**79,79,79,79**,84^g^,84^g^,84^g^,84^g^,84^g^,84^g^,84^g^,84^b^,84^b^,84^b^,84^b^,84^b^,84^b^] | [*15,15*,23,23,**78,78,88**] | [*15*,**88,88**] | 5/33  0 | 4/17  0 | 3/7  0 |
| RECREATION FACILITIES | [84^b^,84^g^,84^g^] | [23,23,**38,38**] | *[15]* |  |  |  | [*15,15*,**72**,84^b^] | [*15,15*,65**,88,88,88,88**] | [*15*,**88,88,88,88**] | 3/7  (+) | 4/11  (+) | 1/6  0 |

x/x = number of positive associations / number of total investigated associations

0 = 0-33% of the findings supporting the association = unrelated evidence

(+) or (-) = 34%–59% of the findings supporting the association = evidence for a possible association

+ or - = 60%–100% of the findings supporting a positive or negative association = convincing evidence

? = variable was investigated in less than 3 studies

Bold: Using objective instruments to determine the physical environment

Italic: Using audit data to determine the physical environment

Underlined: using perceptions to determine the physical environment

b= in boys; g= in girls

# 3. Continent specific correlates of active transportation to school

|  | + | - | 0 | **n/N**  **relation**  **Europe** | n/N  relation North-  America | *n/N*  *relation*  *Australia* | n/N  relation  Asia | total relation |
| --- | --- | --- | --- | --- | --- | --- | --- | --- |
| WALKABILITY | [42,42,56,**67,76,76***,80,86,87*] |  | [**68,69**,*86,87*] | 3/5  + | 3/3  ? | 3/5  + | 0/0  ? | 9/13  + |
| density | [23,42,49,50,54,54,55,60,**73**] |  | [37,40,41,41,42,48,66,**70,77,77**] | 1/4  0 | 8/14  (+) | 0/0  ? | 0/1  ? | 9/19  (+) |
| land use mix diversity | [23,42,45,46,48,51,52,52,61,**75**] | **[29**,37,62,63,63,**77,77**] | [**29**,42,42,43,46,46,60,61,61,61,**75,77,77,77,77**] | 1/10  0 | 9/22  (+) | 0/0  ? | 0/0  ? | 10/32  0 |
| street connectivity | [**15,29**,42,52,54,54,54,54,55,60,**77**,*86*] | [**29**,45,52,52,54,55,61,**74,77,77**] | [**15,15,15,15,15**,23,**29,29***,30,30*,37,37,37,42,52,52,52,53,53,54,60,60,61,**70,77,77,77**,**78**,*86*,*87,87*] | 3/19  0 | 8/28  0 | 1/6  0 | 0/0  ? | 12/53  0 |
| ACCESSIBILITY | [42,46,62,62,63,63,**68,75**,*83,85,86*] | *[85]* | [16,23,*30*,37,37,37,46,**75,***86*] | 2/3  ? | 6/12  (+) | 3/6  (+) | 0/0  ? | 11/21  (+) |
| WALK/CYCLE FACILITIES | [**15,15,15**,16,33,34,34,42,45,45,55,57,57,60,62,62,63,63**,67,77**,*87*] | [**29**,33,77] | [**15,15,15,15,15,15**,23,**29**,*30,30*,36,37,43,43,43,43,43,46,46,46,46,51,60,**64,64,68,70,77,77,77,77,77,77**,*83,83,86,86,87*] | 5/23  0 | 15/31  (+) | 1/8  0 | 0/0  ? | 21/62  0 |
| AESTHETICS | [**15**,36,42,45,46,46,57,*81*] | [**15**,28,34,59,**64,64,73**] | [**15,15,15,15**,23,*30*,37,43,43,43,43,46,46,46,57,57,57,57,57,57,57,60,**64,64,64,64,68,70,75,75**,*81*] | 1/17  0 | 7/29  0 | 1/3  ? | 0/0  ? | 9/49  0 |
| SAFETY | [**29,29**,33-35,45,46,51,57,58,62,63,**67,74,74,76,76,76**,*81,83,86,86,87,87*] | **[15,77,77]** | [**15,15**,16,**29,29,29,29**,34,34,35,45,46,46,53,57,57,59,**66**,**75,75**,**76,76,76,77,77**,*81*] | 8/25  0 | 10/21  (+) | 6/7  + | 0/0  ? | 24/53  (+) |
| crime safety | [45,46,51,57,57,57] | [43] | [23,*30*,36,37,41,42,45,45,46,46,46,57,**68,70**,*85,85,85,87,87,87,87*] | 0/2  ? | 6/18  0 | 0/8  0 | 0/0  ? | 6/28  0 |
| traffic safety | [**29,29**,43,43,43,45,45,45,45,45,46,46,51,52,52,57,57,57,60,60,60,61,63,**64,67,67,67,70,76,76,78**,*82,83,85,85,85,85,85,85,86,86,86,86,86,86,86,86,86,87,87,87,87,87,87,87,87,87,87*][41,57,57,*87*] | [**15**,45,45,58,**78**,*87*] | [**15,15,15,15,15,15**,23,*30,30,30,*36,37,42,43,43,43,43,45,45,45,45,46,46,46,52,55,60,60,60,60,60,60,60,61,**64,66,68,75,75,77,77,77,77,77**,*83,83,85,85,85,85,86,86,86,86,86,86,86,86,87,87,87,87,87,87,87,87,87*][**15,15**,41,52,**77**] | 10/31  0 | 24/54  (+) | 28/55  (+) | 0/0  ? | 62/140  (+) |
| RECREATION FACILITIES | [**15**,16,46,47,60,*81,81*] |  | [**15,15,15,15,15**,23,46,60,**75,75**] | 1/8  ? | 4/7  (+) | 2/2  ? | 0/0  ? | 7/17  (+) |

x/x = number of positive associations / number of total investigated associations

0 = 0-33% of the findings supporting the association = unrelated evidence

(+) or (-) = 34%–59% of the findings supporting the association = evidence for a possible association

+ or - = 60%–100% of the findings supporting a positive or negative association = convincing evidence

? = variable was investigated in less than 3 studies

Bold: European studies

Underlined: American studies

Italic: Australian studies

Standard: Asian studies

# 4. Continent specific correlates of active transportation during leisure time

|  | + | - | 0 | **n/N**  **relation**  **Europe** | n/N  relation  America | *n/N*  *relation*  *Australia* | n/N  relation  Asia | total relation |
| --- | --- | --- | --- | --- | --- | --- | --- | --- |
| WALKABILITY |  | **[69]** | **[69,69]** | 0/3  ? | 0/0  ? | 0/0  ? | 0/0  ? | 0/3  ? |
| density | [38,88,88] | **[69]** | [23,23,38,88,88,88,88,88,88] | 0/1  ? | 1/4  ? | 0/0  ? | 2/8  ? | 3/13  0 |
| land use mix diversity | [23,88] | [88,88] | [23,38,38,38,38,88,88,88,88,88] | 0/0  ? | 1/6  ? | 0/0  ? | 1/8  ? | 2/14  0 |
| street connectivity | [**15**,23,88,88,88,88,88] | **[78,78]** | [**15,15,15,15,15**,23,*31,31,31,31*,38,38,**79,79**,88,88,88] | 1/8  ? | 1/4  ? | 0/6  ? | 5/8  ? | 7/26  0 |
| ACCESSIBILITY | [23,23] |  |  | 0/0  ? | 2/2  ? | 0/0  ? | 0/0  ? | 2/2  ? |
| WALK/CYCLE FACILITIES | [**15,15,15**,23,*31*,88,88,88] | [*15*,88] | [**15,15,15,15,15**,23,*31*,**71**,*79*,88,88,88,88,88,88,88,88,88,88,88,88] | 3/10  ? | 1/2  ? | 1/3  ? | 3/16  ? | 8/31  0 |
| AESTHETICS |  |  | [**15,15,15,15,15,15**,23,23,**72**] | 0/7  ? | 0/2  ? | 0/0  ? | 0/0  ? | 0/9  0 |
| SAFETY |  |  | **[71,72]** | 0/2  ? | 0/0  ? | 0/0  ? | 0/0  ? | 0/2  ? |
| crime safety | [23,**65**] |  | [23,**65**,**71,***84,84,84,84,84,84*] | 1/3  ? | 1/2  ? | 0/6  ? | 0/0  ? | 2/11  0 |
| traffic safety | [**15**,*31*,**71,78,***84,84,84*,88,88,88,88,88] | **[15,15,65,65,65,65,78]** | [**15,15,15,15,15,15**,23,23,*31,31,31,31,31,31,31*,**71,78,78,79,79,79,79,***84,84,84,84,84,84,84,84,84,84,84,84,84***,**88,88,88] | 3/23  0 | 0/2  ? | 4/24  0 | 5/8  ? | 12/57  0 |
| RECREATION FACILITIES | [**15**,23,23,38,38,*84,84,84*] |  | [**15,15,15,15,15**,**65,72,***84*,88,88,88,88,88,88,88,88] | 1/8  0 | 4/4  ? | 3/4  ? | 0/8  ? | 8/24  0 |

x/x = number of positive associations / number of total investigated associations

0 = 0-33% of the findings supporting the association = unrelated evidence

(+) or (-) = 34%–59% of the findings supporting the association = evidence for a possible association

+ or - = 60%–100% of the findings supporting a positive or negative association = convincing evidence

? = less than 3 variables were investigated

Bold: European studies

Underlined: American studies

Italic: Australian studies

Standard: Asian studies
